# Role of Dietary Nutrients in the Modulation of Gut Microbiota: A Narrative Review

**DOI:** 10.3390/nu12020381

**Published:** 2020-01-31

**Authors:** Qi Yang, Qi Liang, Biju Balakrishnan, Damien P Belobrajdic, Qian-Jin Feng, Wei Zhang

**Affiliations:** 1 Center for Marine Drugs, State Key Laboratory of Oncogene and Related Genes, Department of Pharmacy, Renji Hospital, School of Medicine, Shanghai Jiao Tong University, Shanghai 200127, China; qi.yang@flinders.edu.au; 2 Centre for Marine Biopro ducts Development, College of Medicine and Public Health, Flinders University, Adelaide, South Australia 5042, Australia; qi.liang@flinders.edu.au (Q.L.); biju.balakrishnan@flinders.edu.au (B.B.); 3 Shanxi University of Chinese Medicine, Tai Yuan 030619, China; fqj728119@aliyun.com; 4 CSIRO Health and Biosecurity, Adelaide South Australia 5000, Australia; damien.belobrajdic@csiro.au

**Keywords:** micronutrient, macronutrient, gut, microbiome, nutrition, health, diet, review

## Abstract

Understanding how dietary nutrients modulate the gut microbiome is of great interest for the development of food products and eating patterns for combatting the global burden of non-communicable diseases. In this narrative review we assess scientific studies published from 2005 to 2019 that evaluated the effect of micro- and macro-nutrients on the composition of the gut microbiome using in vitro and in vivo models, and human clinical trials. The clinical evidence for micronutrients is less clear and generally lacking. However, preclinical evidence suggests that red wine- and tea-derived polyphenols and vitamin D can modulate potentially beneficial bacteria. Current research shows consistent clinical evidence that dietary fibers, including arabinoxylans, galacto-oligosaccharides, inulin, and oligofructose, promote a range of beneficial bacteria and suppress potentially detrimental species. The preclinical evidence suggests that both the quantity and type of fat modulate both beneficial and potentially detrimental microbes, as well as the Firmicutes/Bacteroides ratio in the gut. Clinical and preclinical studies suggest that the type and amount of proteins in the diet has substantial and differential effects on the gut microbiota. Further clinical investigation of the effect of micronutrients and macronutrients on the microbiome and metabolome is warranted, along with understanding how this influences host health.

## 1. Introduction

Over the past two decades a plethora of studies have identified broad-spanning associated links between the gut microbiota and systemic health and disease risk [1,2,3]. Earlier, it was estimated that the human body harbors about 10^14–^10^15^ microbial cells [4,5,6], which is thought to outnumber host cells in the human body by a ratio of at least 10:1 [7]. This was refuted recently, with an estimate that the ratio is much closer to 1:1 [6]. Importantly, the human microbiome is far more diverse than that of human cells, which adds to the complexity in trying to understand how the microbes and their metabolites effect health and modulate the development of disease [8]. This challenged the researchers to explore the roles of the human microbiota and their potential applications in managing human health and disease conditions.

The large intestine is the predominant location where microbes are present in the human gut and our understanding of the composition, interaction, and functions of these gut microbes [9] continues to develop as a result of advancement in major initiatives that have categorized the microbiome [1,10,11,12,13]. The human gut microbiome is highly dynamic during the various stages of human development and has been implicated in a variety of health and disease conditions. There are many factors that contribute to shaping the gut microbial colonization, growth, composition, and diversity. The major factors that impact microbial colonization and diversity include age [14], genetics [15,16,17], mode of delivery at birth [18,19], method of feeding in infants [20,21], medications (e.g., antibiotics), [22,23] geographical location [24], and the diet [25]. Metagenomics studies in a Dutch population have shown associations between gut microbiota and 126 exogenous factors, 31 intrinsic factors, 12 diseases, 19 drug groups, 4 smoking categories, and 60 dietary factors [26]. The responses of the gut microbiota to sensitive factors are considered to be a valuable tool to exploit and develop new strategies to promote human health. Among these influential factors, dietary factors, including micro- and macro-nutrients, are the most influential in shaping and modulating the human gut microbiota [27].

Interactions between the intestinal microbiota and the host are crucial to understand the role of the microbiota in biological processes, and how they contribute to health and the development of disease. Microbial diversity measures the distribution of different species on the community, the level of which is reduced during gut dysbiosis, and a richness of species indicates a “healthy gut” [28,29,30]. Lower bacterial diversity was reported in people suffering from inflammatory bowel disease, Type 2 diabetes (T2DM), and Coeliac disease. Recent studies have identified the dominance of some groups of gut microbes that are associated with a good health outcome [28,29,30], and these microbes are presented in this review as “potential beneficial microbes”, that include major species from the genera *Bifidobacterium, Lactobacillus, Akkermansia, Fecalibacterium, Eubacterium, Roseburia, Ruminococcus,* and *Blautia*. Studies have also reported the abundance of specific bacteria that could potentially contribute to the development or progression of major non-communicable diseases and these microbes are presented in this review as “potential detrimental microbes”, that include some species from the genera *Clostridium, Enterobacter, Enterococcus, Bacteoidetes,* and *Ruminococcus* [31,32,33]. Both human and animal studies report that an increase in the Firmicutes to Bacteroidetes ratio (F/B ratio) is associated with obese/lean phenotypes and may modulate energy balance [34,35].

The aim of this narrative review was to assess scientific studies that evaluated the effect of micro- and macro-nutrients on the composition of the gut microbiome using in vitro and in vivo models, and human clinical trials. Scientific studies published between 2005 and 2019 in the databases of PubMed, Scopus, and Web of Science were identified using specific search terms (Supplementary Table S1). Publications that did not specifically quantify changes in gut microbiota were excluded, resulting in a total of 213 articles that were selected for preparing this review. The major findings relating to the microbiome, including the shifts in potentially beneficial and detrimental gut microbiota, the F/B ratio, and microbial diversity, are discussed in this review. The studies reviewed included in vitro and in vivo models and human clinical trials (in that order whenever available) and were separated based on micro- and macro-nutrients. 

## 2. Role of Dietary Micronutrients in Modulating Gut Microbiota

### 2.1. Polyphenols

Polyphenols, such as flavonoids, phenolic acids, stilbenes, and lignans [36] from fruits, vegetables, cereals, tea, coffee, and wine [37], have attracted research interest due to their potential anti-oxidative, anti-inflammatory, and anti-carcinogenic effects [38]. In vitro studies suggest that polyphenols could modulate human gut microbiota by inhibiting potential pathogenic organisms (such as *Helicobacter pylori*, *Staphylococcus* sp.) and favoring the growth of potential beneficial members, including *Lactobacillus* and *Bifidobacteria* [39,40]. Animal and clinical trials show that polyphenols can modulate gut microbes, microbial diversity, and the ratio of Firmicutes to Bacteroidetes (F/B) [41,42,43,44]. These and other studies have suggested that the prebiotic-like activities of polyphenols are the major reason behind the health benefits imparted by polyphenols in humans [45]. Herein, we discuss in vitro, animal, and clinical trial data that evaluated the effect of polyphenols on gut microbes, microbial diversity, and F/B ratio, and the major findings are summarized in Table 1.

In vitro studies have shown that flavonoids such as anthocyanins, phenolic acids (epicatechins, *p*-coumaric acid, and *o*-coumaric acid), and other polyphenols like quercetin, rutin, chlorogenic acid, and caffeic acid could increase the abundance of beneficial gut microbiota, such as *Bifidobacterium* and *Lactobacillus,* and decrease the number of potential pathogenic bacterial colonization in the human gut [39,40,46,47]. Catechins have also been shown to stimulate the growth of the *Clostridium coccoides*-*Eubacterium rectale* group, *Bifidobacterium* sp., and *Escherichia coli* and inhibit the growth of a potential pathogenic organism (*Clostridium hystolyticum* group) [48]. Fermentation of polyphenols increases the abundance of beneficial microbiota such as *Bifidobacteria* and reduces the F/B ratio by promoting the growth of specific members of the phylum *Bacteroidetes* [49]. It was proposed that the bio-transformed polyphenols promoted the production of short chain fatty acids (SCFAs), which are well recognized as playing an important role in gut and metabolic health [50]. By utilizing agar plate assays, Naringenin, a flavone present in citrus fruits, modulated the growth and genetic regulation of gut commensal bacteria [51]. Ethyl acetate/water polyphenol extract of Yunnan Chinese tea was reported to inhibit the growth of unfavorable bacteria species (*Clostridium* and *Bacteroides*) in vitro [52].

Preclinical studies have shown consistent evidence that a range of different polyphenols improve various measures of health by modulating gut microbiota. In animals fed a High-Fat Diet (HFD) or control background diets, polyphenols including grape [53], pomegranate peel [54], red wine [55], and green tea [56] have been shown to increase the abundance of fecal *Bifidobacteria* and *Lactobacilli*. Axling et al. (2012) [56] also found that green tea powder along with *Lactobacillus plantarum* could significantly increase bacterial diversity and increase the abundance of *Lactobacillus* and *Akkermansia* in the colon of mice [56]. The fecal microbiome of rats fed with blackberry anthocyanin rich extracts were found to have increased abundance of *Psudoflavonifractor* when compared to the standard control diet, whereas in the presence of an HFD, *Akkermansia* numbers were restored to the levels seen in a low-fat diet [57]. The anthocyanin extracts also increased the abundance of *Oscillobacter* in rats fed a control diet or HFD [57]. Phloridzin administration was found to increase the abundance, thereby restoring the beneficial species, of *Akkermansia* in a diabetic mouse model [58]. This microbial modulation and associated SCFA production are thought to reduce the lipopolysaccharide (LPS) content in Phloidzin-treated mice, and the authors suggested this as a potential mechanism behind the improvement seen in diabetic mice after the administration of Phloridzin. Grape polyphenols have been shown to increase *Bifidobacterium* numbers in rats of control group [59] and *Lactobacillus* numbers in broiler chickens [60]. A reduction in F/B ratio was demonstrated by feeding polyphenols to an HFD-induced rat model [41,42,43,44]. In a colitis mouse model, when animals were fed high levels of curcumin (standard rodent diet containing 0.2% w/w of nanoparticle curcumin), there was an increase in butyrate-producing bacteria, viz. *Clostridium* sp. cluster IV and XIVa, that activated T regulatory cells-mediated suppression of colitis in this model [61]. Genistein supplemented corn oil diet in germ-free humanized mice that received fecal transplant from breast cancer patients increased the abundance of beneficial gut microbiome in their gut [62]. The bacterial species that were increased after genistein feed in these mice included *Eubacterium dolichum*, *Lactococcus lactis*, *Akkermansia municiphila*, *Ruminococcus torques,* and *Clostridium hathewayi,* and the bacterial species that were significantly lower than that of control group included *Bacteroides eggerthii* and *Bacteroides ovatus* [62]. Based on their observation in this study the authors further hypothesized that this epigenetic regulation might have played a role in genistein-fed mice to reduce their tumor size and latency [62].

A small number of clinical trials have reported changes in the composition of the microbiome following the consumption of polyphenol-rich foods, that include cocoa, red wine, green tea, and vegetable/fruit. Cocoa-derived polyphenols (494 mg/day) when consumed for four weeks were shown to significantly increase the fecal abundance of *Bifidobacterium* and *Lactobacillus* compared to a low polyphenol diet (23 mg/day) [63]. This prebiotic-like effect was further confirmed by conducting in vitro fermentation analysis on fecal samples from the same study’s participants [63]. In another study, polyphenols from red wine increased the number of *Bifidobacteria* and *Lactobacilli* in the feces of obese individuals, which correlated with an improvement in metabolic syndrome markers, including blood pressure, plasma glucose level, and plasma lipid profile [64]. In addition, red wine polyphenols increased the number of butyrate-producing microbes *Fecalibacterium prausnitzii* and *Roseburia* [64] in feces. Moreover, in a similar study, the modulation of beneficial microbiota such as *Bifidobacterium* sp. *Eubacterium rectale*, *Bacteroides uniformis*, *Prevotella* sp., *Blautia coccoides*, and *Eggerthella lenta* was also reported for participants consuming red wine polyphenols [65]. The consumption of a vegetable/fruit juice-based diet on three consecutive days could reduce the F/B ratio of healthy adults [66]. Green tea consumption increased the proportion of the *Bifidobacterium* sp. along with bacterial metabolite production [67]. In a study involving an adult population (*n* = 1044), it was reported that Diadzin intake could increase the equol-producing bacteria, such as *Asaccharobacter celatus* and *Slackia isoflavoniconvertens,* in the gut [68]. Findings from a study of Mayta-apaza et al. [69] suggested that the background diet and individual’s microbiome may be a key determinant of the gut microbial metabolism of polyphenols. This study indicated that only when individuals had low Bacteroides numbers was polyphenol supplementation effective in increasing *Bifidobacterium* numbers. Alternatively, individuals with high numbers of Bacteroides, indicative of a low carbohydrates and fiber diet, western-style eating pattern, had a lower ability to metabolize polyphenols, thereby reducing the bioavailability and potential health benefits of polyphenols. Tart cherry juice consumption significantly changed the microbiome, and interestingly, it increased *Bacteroides*, *Parabacteroides,* and *Alistipes* in individuals with high levels of *Bacteroides* at baseline, and the opposite was observed for those with a low level of *Bacteroides* [70]. Pomegranate extract could increase beneficial microbiota *Akkermansia*, *Lactobacillus*, and *Prevotella* [71]. A study by Most et al. suggested that men and women may metabolize polyphenols differently. In that study, the supplementation of either epigallocatechin-3-gallate and resveratrol modulated gut microbiota in overweight men, with an increase in F/B ratio, but a similar association was not observed in overweight women [72]. Although studies have shown an influence of gender and body mass index on intestinal microbiota [73], the mechanisms for this difference in gender response in metabolizing polyphenols is not well understood.

It is evident that the beneficial health effects of polyphenols could be partly due to their ability to modulate gut microbiota. Major groups of polyphenols assayed in both in vitro and preclinical studies have shown their ability to modulate the gut microbiota to a beneficial pool characterized by the abundance of *Bifidobacterium, Lactobacillus, Akkermansia,* and *Fecalibacterium* sp. The beneficial mechanisms observed in those studies were mainly attributed to the production of SCFAs and other bacterial metabolites that contributed towards positive changes in gut health and reducing the inflammatory process, thereby improving systemic disease status. Although there are only limited clinical trials that have specifically evaluated the health effects of polyphenols, the results are highly promising, and the microbial modulation observed in these studies mimics that of preclinical studies. Based on these observations it is highly recommended to conduct more studies to explore the actions of specific polyphenols in modulating human gut microbiota, and thereby their effects on improving/preventing metabolic diseases and cancer.

### 2.2. Vitamins

Vitamins are organic compounds that are essential in very small amounts for supporting normal physiological function. They often serve a variety of roles in the body—one of the most important being their roles as cofactors for enzymes. The diet is the primary source of vitamins, as our bodies cannot synthesize them to meet our daily needs, but certain vitamins, notably vitamin K, and B group vitamins, are synthesized by gut microbiota [74]. When vitamins are deficient, chronic health conditions can be created or exacerbated, and it is common for people to consume individual or multiple vitamin supplements, which can provide very high doses of specific vitamins. Subsequently, minimal absorption of these vitamins in the upper gut can modulate the abundance and diversity of the gut microbiota. The role of dietary vitamins in the modulation of gut microbiota using both animal models and clinical trials are summarized in Table 2.

Vitamin A, a fat soluble vitamin, has been indicated as an adjuvant therapy for infectious diseases [75,76,77,78], and has a potential adjunct therapeutic effect on children with autism spectrum of disorders (ASD) [79], possibly by altering gut microbiota. The diversity of gut microbiota and key phylotypes differed significantly in children with persistent diarrhea, whose vitamin A levels differed considerably [80]. A decrease in butyrate-producing bacteria (*Escherichia coli* and *Clostridium butyricum*) and an increase in opportunistic pathogens (*Enterococcus*) might have partially caused the reduced diversity in the vitamin A deficiency group [80]. Vitamin A supplementation in the form of retinoic acid in a murine model could inhibit Murine Norovirus replication [75,76]. In this study, the researchers demonstrated that the administration of retinoic acid (physiologically active metabolite of vitamin A) significantly increased the abundance of *Lactobacillus* sp. during a norovirus infection. In an in vitro model, *Lactobacillus* showed antiviral activity against norovirus, and based on these data, the authors hypothesized that the abundance of *Lactobacillus* in the gut was partially responsible for norovirus inhibition [75,76]. It was also shown that retinoic acid administration increased the abundance of *Allobaculum*, *Aggregatibacter*, *Bifidobacterium*, *Dialister*, and *Enhydrobacter* [75,76]. Epidemiological studies have shown that the rate and clinical symptoms of norovirus infection decrease significantly with sufficient vitamin A supplementation [77]. In addition, vitamin A supplementation was reported to reduce both mortality and morbidity associated with infectious gastrointestinal diseases [78]. In a pilot study, vitamin A administration significantly increased both Bacteroidetes and Bacteroidales populations and reduced F/B ratios in children with ASD [79]. It was suggested that the supplementation of vitamin A to children with ASD improved the condition, possibly by restoring the Bacteroidetes/Bacteroidales population in their gut. Moreover, better vitamin A status in infancy may influence health, both in infancy and later in life, by promoting the establishment of a healthy microbiota. Huda et al. (2019) [81] reported that the supplementation of infants in early (6–15 week) or late (2 year) infancy with 50,000 IU vitamin A could increase the abundance of *Bifidobacterium* and *Akkermansia* in their feces, but did not affect the abundance of Proteobacteria.

The B-group vitamins are a collection of eight water-soluble vitamins essential for various metabolic processes. Even though B-group vitamins are found in many foods (e.g., animal-based foods, leafy green vegetables, beans, and peas), they are easily reduced, particularly by alcohol and cooking. Some B vitamins have been shown to promote bacterial colonization, modulate bacterial virulence, and take part in pathogen interactions with the host through modification of the host defense [82]. For example, supplementation of vitamin B12 was found to enhance the colonization of *Bacteroides thetaiotaomicron* in the gut of an experimental gnotobiotic mice model [83]. Moreover, vitamin B12 is also essential for some enteropathogens to utilize ethanolamine, which enhances *Salmonella typhimurium* growth and its virulence gene expression, as demonstrated in in vitro and in vivo models [84,85]. At the same time, all Fusobacteria and over 90% of Bacteroidetes were predicted to be the producers of B12 by biosynthesis [86]. Similarly, vitamin B6 could be produced by gut microbiota, Bacteroidetes [86], and is mainly utilized as a co-factor for many biological reactions associated with host immune response. B6 deficiency could induce atrophy of lymphoid organs, marked reduction in lymphocyte numbers, and impaired antibody responses and IL-2 production in a clinical study [82,87]. Animal experimentation and human clinical trials further demonstrated that vitamin B6 promotes the growth of *Bacteroides*, which was mediated either by modulating the host immune system or by interfering with the growth or expression of virulence factors of *Salmonella typhimurium* [87]. On the other hand, gut dysbiosis in a murine model could reduce the luminal vitamin B6 level and lead to colonization in the gut by enteropathogenic strains of *Salmonella* sp. [88]. From these preliminary studies it should be cautioned that vitamin B3 and B6 supplementation could lead to an abundance of deleterious/potentially pathogenic species in the gut, and hence could lead to unwanted side effects.

Vitamin C is the most important water-soluble antioxidant in the human body. In contrast to other water-soluble vitamins, vitamin C cannot be synthesized de novo in humans and has to be obtained from dietary sources (fruits and vegetables) via intestinal absorption [89]. The redox state could strongly modulate the gut microbiota. It was found that vitamin C intake was positively correlated with Firmicutes and its lower taxa (i.e., *Clostridium*) and negatively associated with Bacteroidetes in an assessment of a small group of free-living adults with stable cystic fibrosis [90]. Wilson et al. (2018) [91] investigated the effect of consuming two SunGold kiwifruit per day over 12 weeks on vitamin C status and fecal microbiota composition in people with prediabetes. Analysis showed an increase in the relative abundance of uncultivated members of the bacterial family Coriobacteriaceae; however, these changes were small and were not clinically significant. An animal study conducted on early-weaned piglets confirmed the antioxidant capacity of vitamin C in scavenging free radicals and restoring the gut microbiota microenvironment, increasing *Lactobacillus* and *Bifidobacterium* counts, and decreasing *E. coli* counts in the gut environment [92].

Clinical trials involving vitamin D supplementation have shown positive health outcomes to help people maintain health and prevent chronic diseases, and subsequent changes in the microbiota may be a significant mechanism [93,94]. As a fat-soluble vitamin, vitamin D is thought to participate in the process of neurotransmitter synthesis and calcium balance, protecting nerve cells by its antioxidant effects [95]. In a cohort study involving 56,366 American women aged 50 to 79 years it was shown that high levels of Vitamin D intake can significantly reduce the risk of depression [96]. Mounting evidence suggests that the alteration of gut microbiota by vitamin D could be the reason behind this. A randomized control trial [97] showed that weekly supplementation of vitamin D, (50,000 ergocalciferol IU) over 12 months increased the fecal levels of SCFA and abundance of SCFA-producing genera, such as *Ruminococcus*, *Fecalibacterium*, and *Dialister*. Vitamin D3 supplementation was also reported to have a positive impact on the gut microbiota in cystic fibrosis patients by increasing the beneficial bacteria of the genus *Lactococcus* and decreasing the abundance of *Veillonella* at genus level and *Erysipelotrichaceae* at the family level, of which many members were found to be potential pathogens [98]. Vitamin D administration could prevent or even treat various malignant tumors [99,100] and gastrointestinal inflammatory diseases [101,102]. For example, supplementation with vitamin D3 significantly decreased the relative abundance of gamma-proteobacteria and increased bacterial richness in humans [103]. In this study, vitamin D3 modulated the gut microbiome of the upper GI tract, which could explain its positive influence on gastrointestinal diseases, such as inflammatory bowel disease or bacterial infections. Vitamin D has immunomodulatory properties and hence could potentially affect the microbial colonization of the intestinal tract [104,105,106]. In a study of Garg et al. (2018) [107], the effect of vitamin D replacement in vitamin D-deficient patients with and without ulcerative colitis (UC) was investigated on inflammation and fecal microbiota. Vitamin D supplementation (one dose of 40,000 IU once weekly for eight weeks) was found to be associated with reduced intestinal inflammation in patients with active UC, with a concomitant increase in Enterobacteriaceae but no change in overall fecal microbial diversity. In addition, vitamin D also showed a specific influence on the bacterial communities in Crohn’s disease (CD), but not in healthy controls [108]. In this study, the microbiota of the members of the genera *Alistipes, Barnesiella*, unclassified Porphyromonadaceae (both Actinobacteria), *Roseburia, Anaerotruncus, Subdoligranulum* and an unclassified Ruminococcaceae (all Firmicutes) were increased significantly after administration of vitamin D for one week in CD patients. This clearly suggests that the administration of vitamin D may have a positive effect on CD by modulating the intestinal bacterial composition and also by increasing the abundance of potential beneficial bacterial strains. Furthermore, maternal administration of vitamin D during pregnancy had a negative linear association with *Bifidobacterium* sp. and a positive association with the *Bacteroides fragilis* group in infants, suggesting that the prenatal vitamin D administration had an effect on bacterial diversity in the infants [109]. Reduced abundance of *Clostridium difficile* was associated with vitamin D supplementation of breast-fed infants whose mothers were more likely to adhere to a lifestyle with regards to dietary habits as vegetarians, or organic/macrobiotic diets. These data suggest that pre/postnatal vitamin D exposure influences the abundance of several key bacterial taxa within the infant microbiota, thereby leading to the development of health beneficial/detrimental microbiota in infant gut [109]. Luthold et al. (2017) [110] examined the association between vitamin D intake and circulating levels of 25(OH)D with gut microbiota composition, inflammatory markers, and biochemical profile in healthy individuals. *Prevotella* was more abundant, while *Haemophilus* and *Veillonella* were less abundant in the subset with the highest vitamin D intake (≥ 10 μg/day). In addition, abundances of *Coprococcus* and *Bifdobacterium* were statistically inversely correlated with 25(OH)D, while vitamin D deficiency could significantly affect the fecal microbiota of healthy adults, as well as play an important role during the progression of hypertension. For example, healthy individuals who received less than 50 nmol/L 25(OH)D had a lower abundance of genus *Coprococcus* and higher abundance of genus *Ruminococcus* compared to those who received more than 75 nmol/L 25(OH)D [111]. The study of Zuo et al. (2019) [112] reported that vitamin D3 was positively correlated with health beneficial bacterial genera, including *Subdoligranulum*, *Ruminiclostridium*, *Intestinimonas*, *Pseudoflavonifractor*, *Paenibacillus*, and *Marvinbryantia*, which were thought to have antihypertensive function.

Vitamin E, well recognized for its antioxidant effects, is commonly found in a range of food products, including wheat germ oil, extra virgin olive oil, hazelnuts, peanuts, fish, oysters, eggs, and butter. It has been shown to protect against mucosal tissue damage in chemical-induced colitis models [113,114]. It has also been demonstrated that natural antioxidants may regulate the gut microbiota composition by scavenging excessive free radicals and supporting the cellular and humoral immune responses [115]. Recent findings in a mice model of ileal pouchitis, an antioxidant diet, enriched in vitamins E, selenium, and retinoic acid, may reshape the gut microbial community toward an anti-inflammatory profile, mitigating mucosal inflammation. This capacity appears to be mediated by an increase in the relative percentage of Bacteroidetes and a decrease in Firmicutes at the phylum level, with an overall increase in alpha-diversity (Shannon diversity index) [116]. Another mice model study compared the gut microbiota composition between groups with low vitamin E (0.06 mg/20 g of the body weight) and high vitamin E (0.18 mg/20 g) [117]. A reduced ratio of Firmicutes to Bacteroidetes was found in high-level consumption of vitamin E compared to control and low-level consumption. This evidence was confirmed by a human study conducted in pregnant women in the second trimester, which showed that a higher intake of vitamin E was associated with a decrease in Proteobacteria and Firmicutes and an increase in Bacteroidetes [118]. Moreover, a recent study conducted on iron-deficient infants and toddlers showed an increase of the relative abundance of the genus *Roseburia* (phylum Firmicutes), a butyrate producer, in the group supplemented with iron and vitamin E compared to that in the only iron-supplemented group [119].

It is evident from the above discussion that there is a high level of interactions between the vitamins and gut microbiota in that some vitamins are produced by the gut microbiota and others are responsible for modulating beneficial/detrimental species based on the concentration within the microenvironment. Both preclinical and clinical trials have shown the ability of vitamin A in modulating health-beneficial microbes of the genera *Bifidobacterium, Lactobacillus,* and *Akkermansia*. The F/B restorative function of vitamin A in ASD patients is very interesting and this demands further studies for its use in combinational therapy for patients suffering from ASD. Interestingly, some B complex vitamins are produced by gut commensals and some of these vitamins participate in enhancing the virulence/colonization of potential pathogenic microbes. Vitamin C, D, and E supplementation could modulate health-beneficial microbiota, especially beneficial species from the genera *Bifidobacterium* and *Lactobacillus.* Vitamin D and E also modulate health-beneficial microbes of the genera *Roseburia*. Moreover, vitamin D and E may also reduce F/B ratio. Taken together, these studies suggest that the supplementation of vitamins could modulate gut microbiota. However, the modulation depends on the vitamin level in the host, and hence further clinical trials are warranted to prevent any adverse reaction by “over supplementation” of vitamins.

### 2.3. Minerals and Trace Elements

Minerals and trace elements are essential micronutrients for human metabolism and perform active interaction with the gut microbiome [120,121]. Both nutritional deficiency and an excess of minerals and trace elements are responsible for various diseases in humans. The role of trace element excess or deficiency in modulating gut microbiota is an emerging field and the major findings from the published articles are summarized in Table 2.

Epidemiological data suggest that high calcium intake is associated with a lower prevalence of obesity [122]. It is suggested that a high intake of calcium leads to changes in the gut microbiota, which are associated with a lean phenotype [122,123]. In a healthy human intervention study, dietary intake of 1000 mg calcium per day for eight weeks resulted in a higher *Clostridium* XVIII in the fecal samples of men [124]. As this bacteria species is an unculturable bacteria it has not been classified as beneficial or detrimental, but calcium in combination with phosphorous (500 mg Ca and 1000 mg p) could lead to an abundance of butyrate producers in the feces. In an 18-month high-fat fed mouse study, calcium supplementation (5.25 g/kg of calcium) increased the microbial diversity and the number of *Ruminococcaceae* and *Akkermansia* in the fecal microbiome of these animals [125]. A multigenerational study was conducted to evaluate how an imbalance in maternal calcium promotes body weight gain in their offspring [107]. Excess dietary calcium (12 g/kg) in the maternal diet was associated with a decrease in *Verrucomicrobia* in the offspring’s gut, and insufficient calcium in the maternal diet (2.5 g/kg) was associated with increased F/B ratio in the offspring [126]. In a nutritional intervention of shorter duration (54 days), high calcium supplementation (12 g/kg) modulated gut microbiota in a prebiotic manner by increasing the number of *Bifidobacterium* sp., and increasing *Bacteroides/Prevotella* ratio in the cecal sample of an HFD mouse model. The number of *Bifidobacterium* sp. in this study was negatively correlated with the plasma LPS level, indicating the reduction in LPS producers in the gut microbial pool [127]. 

Magnesium deficiency is associated with an increased incidence of chronic disease [128], but the evidence for the role of the microbiome in this association is not clear. It was reported previously that four days of magnesium deficiency could reduce the bifidobacterial content in mouse cecum, but with prolonged magnesium deficiency (three weeks) there was an increase in the intestinal content of bifidobacteria and lactobacilli [129]. In contrast, it was reported that six weeks of magnesium deficiency could significantly alter the gut microbiota (only Principal Component Analysis (PCA) analysis results were shown) which may be associated with altered anxiety-like behavior in mice [130,131]. Furthermore, a decrease in the gut microbial diversity was reported with dietary magnesium deficiency [130,131] and an increase in the gut microbial diversity in adult male rats was reported with a magnesium-rich marine mineral blend [132]. More research is needed to further identify the association between magnesium deficiency or magnesium supplementation and the gut microbiota.

Iron supplementation is a common strategy to correct iron-deficiency in clinical settings. However, still no consistent conclusion has been achieved for the effect of iron supplementation on gut microbiota [133]. Both preclinical and clinical studies have shown consistent reductions in the abundance of beneficial microbes and an increased abundance of detrimental microbes after supplementation with Iron. Iron supplementation to anemic Kenyan infants [134,135] and Ivorian children [136] has been suggested to cause gut dysbiosis and inflammation as a result of an increased abundance of pathogenic bacteria and a reduction in beneficial microbiota. In a randomized controlled trial [137], consumption of iron-fortified cereal for 2–4 weeks could lead to a reduction in the median relative abundance of bacteria of the family bifidobacteriacea (declined from 51% to 37%) and an increase in bacteria of the order bacteroidetes (from 5% to 14%) in the stool samples of infants. Furthermore, there was no bacterial richness following the intervention. In a randomized placebo-controlled clinical trial the iron supplementation (50 mg/day, 4 d/week for 38 weeks) did not significantly modify the concentrations of dominant bacterial groups (neither beneficial nor pathogenic) in the gut of children living in rural South Africa. This is in stark contrast to the Ivorian children [136] and may be due to environmental differences between these two cohorts. The former group was living in poverty with poor-quality water and diet and hence had higher pathogenic bacterial load in their gut than the latter group, who had a lower concentration of pathogenic bacteria due to the consumption of good quality water and diet [138]. In a comparative study on infants, two different doses of iron supplementation yielded varying effects on the abundance of beneficial microbiota. In comparison, iron supplementation at a higher dose (6.4 mg/day) significantly reduced the abundance of *Bifidobacterium* levels compared to iron supplementation at a lower dose (1.2 mg/day) in infant gut microbiota. However, the opposite was observed for an abundance of *Lactobacillus,* with an increased abundance with this higher dose than the lower dose [139]. Studies on experimental animals have also demonstrated that excess iron could cause intestinal dysbiosis, which leads to an increase in bacteria of the genera *Defluviitaleaceae*, *Ruminococcaceae*, and *Coprococcus* and a reduction in some members of family *Lachnospiraceae* and genus *Allobaculum* [140]. The same was reflected in in vitro fermentation studies, where it was shown that increased iron concentrations could decrease the number of commensal bacteria, increase the number of toxic metabolites and increase the virulence of pathogenic bacteria [141,142]. It was also reported that iron levels up to 60 mg/d did not significantly alter the composition of the fecal microbiome at any taxonomic level in overweight and obese women in early pregnancy [143]. In contrast, in a cross sectional study, consumption of a diet containing a significantly higher iron concentration could lead to an increase in *Bifidobacterium* levels in Japanese women [144]. However, the results of this study should be interpreted with caution as the diet contained micro and macronutrients other than iron and the bifidogenic response might have come from other ingredients in the diet.

The gut microbiota also responds differently depending upon the chemical form of the dietary iron supplemented. Ferric ethylenediaminetetraacetic acid, compared to ferrous formulations, exacerbated dextran sulfate sodium (DSS)-induced colitis in mice by reducing the abundance of *Roseburia* sp. [145]. In comparison, non-heme iron interventions could increase the abundance of Firmicutes in mice, while heme iron decreased the abundance of Firmicutes, along with decreasing overall microbial diversity, and increased the abundance of Proteobacteria [146,147,148].

Additionally, the ways of administration of iron have also been reported to have different influences on gut microbiota. Lee et al. (2017) [149] reported that oral administration of iron resulted in a lower abundance of *Fecalibacterium prausnitzii, Ruminococcus bromii, Collinsella aerofaciens,* and *Dorea* when compared with that of the intravenous administration. It was also reported that the administration of iron as drops at the required standard dose could lead to decreased relative abundance of lactobacilli, and potentially increase susceptibility to bacterial infection [139].

Phosphorus is the second most abundant inorganic element in the body and plays an important role in the maintenance of blood systemic acid balance [150]. The recommended intake for phosphorus is 700–1000 mg for adults, but intake levels are commonly exceeded when processed foods such as baked goods and sugar-sweetened beverages are consumed. A study on broiler chickens showed that phosphorus supplementation increased the abundance of the butyrate-producing bacteria Fecalibacterium and Pseudoflavonifractor in the cecal digesta [151]. A dietary intervention study in human with phosphorus supplementation (1000 mg/day) showed that fecal microbial diversity improved and SCFA concentration increased [124]. However, further clinical studies are needed to investigate the actions of magnesium on gut microbial modulation before any conclusions can be made.

Zinc is an essential micronutrient for maintaining epithelial integrity, possibly by modulating the beneficial gut microbiota [152]. Chronic zinc deficiency in broiler chickens reshapes the gut microbiome, with a significant increase in the abundance of Proteobacteria and a decrease in the abundance of Firmicutes [153]. Animal experimentation has suggested that zinc supplementation (120 mg/kg) [154] in a “Salmonella typhimurium-challenged broiler” model increased the number of beneficial bacteria, such as *Lactobacillus* sp., while reducing the number of detrimental bacteria, including *Salmonella* sp. In mice, however, excess dietary zinc alters the diversity and structure of the gut microbiota. In particular, the genera of *Turicibacter* Operational Taxonomic Unit (OTU) 2 and *Clostridium* OTU 11 decreased and the genera of *Enterococcus* OTU 4 and *Clostridium* XI OTU 3 increased. Furthermore, excess zinc rendered the gut microbiota vulnerable to low-level perturbations and decreased the threshold of antibiotics needed to diminish colonization resistance to the nosocomial pathogen *Clostridium difficile* [155]. Chronic zinc deficiency in broiler chickens reshapes the gut microbiome, with a significant increase in the abundance of Proteobacteria and a decrease in the abundance of Firmicutes [153]. However, the clinical data on the modulation of gut microbiota by dietary zinc in humans are lacking.

A deficiency and excess of selenium is related to health conditions, such as increased mortality, type 2 diabetes, and cancer risk [156]; however, there is limited information regarding the effects on the gut microbiota. Dietary selenium supplementation at a dose range of 0.1 ug/g to 2.25 ug/g in mice was shown to increase the microbial diversity [157]. In another mice study, the supplementation of selenium at a concentration of 0.4 mg/kg led to an increase in the abundance of *Akkermansia* and *Turicibacter*, and a decrease in the abundance of *Dorea* and *Mucispirillum* [158].

Although the evidence is limited to only one animal study, it suggests that iodine supplementation is dependent on the levels of fat in the diet and results in differential effects on the gut microbiome [159]. Iodine supplementation in an HFD mouse model improved the thyroid hormone status, but resulted in a gut dysbiosis characterized by an increased abundance of pathogenic microbes and a depletion of beneficial microbes, such as *Fecalibacterium prausnizii* [159]. Alternatively, when the diet was low in fat, the same dose of iodine had beneficial effects on gut microbiota by increasing *Bifidobacterium*, *Lactobacillus*, *Fecalibacterium*, and *Allobaculuum* in the control group [159].

In summary, there is limited evidence supporting specific mechanisms whereby minerals and trace elements modulate the gut microbiome. However, the studies published to date mostly evaluated changes in the microbiome following micronutrient deficiency and supplementation of minerals and trace elements. Calcium supplementation has been shown to modulate *Akkermansia*, *Bifidobcterium,* and *Ruminococcacea* and the ratio of *Bactereoidetes*/*Prevotella* in experimental animals. The sufficiency/deficiency of Mg in modulating gut microbiome is not clear, as acute deficiency in a mice model seems to modulate health-beneficial gut microbiota. Evidence is inconsistent in iron supplementation and most of the studies show an increase in deleterious microbes and reduction in the abundance of beneficial microbes such as *Bifidobacterium* in human infants, but no influence of iron supplementation on microbiota also been reported in clinical trials. Interestingly, chemical form and route of administration of iron seems to be important in modulating gut microbiome. Limited research has shown that Phosphorus supplementation affects SCFAs, warranting further preclinical/clinical trials to arrive at a conclusion. Zinc supplementation reduced deleterious microbes and increased beneficial microbes in preclinical studies. Selenium supplementation increased the gut microbial diversity and positively modulated health beneficial microbes, such as *Akkermansia* and *Turicibacter,* and negatively modulated the deleterious microbes, such as *Dorea* and *Mucispirillum,* in a mice model. Iodine supplementation has resulted in gut dysbiosis and also reduced the abundance of health beneficial microbes, such as *Fecalibacterium,* in a mice model. There is a clear lack of preclinical/human intervention studies exploring the role of specific minerals and trace elements on modulating gut microbiota, and hence a thorough study in this area is mandated. 

## 3. The role of Dietary Macronutrients in Modulating Gut Microbiota 

### 3.1. Carbohydrates

Carbohydrates are the predominant energy source for the human body and play an important role in modulating and shaping the gut microbiota. Here, we summarize the evidence for how different types of dietary carbohydrates modulate the microbiota at their genus level, the F/B ratio, and the microbial community diversity (Table 3).

Plant-derived carbohydrates that escape digestion in the upper digestive tract are classified as dietary fiber and their structure, along with other undigested nutrients, influences the extent to which they are fermented by the large intestinal microbes. In animal models, the Western-style diet, with relatively lower fiber content, has been shown to reduce the abundance of *Bifidobacterium*, and the diversity of gut microbiota [160]. A chronic lack of dietary fiber could reduce the diversity of gut microbiota [161].A pre-hispanic Mexican diet (high fiber) has been shown to alleviate gut dysbiosis in the rats fed with a sucrose-enriched high-fat diet, as evidenced by reducing the Firmicutes to Bacteroidetes ratio (F/B ratio) and increasing the abundance of *Lactobacillus* sp. [162]. In a preclinical trial, humanized mice were fed a diet rich in fiber and later introduced a feed with low-quality fiber to perturbate their gut microbiome [15]. However, re-introduction of fiber by feeding a plant polysaccharide-rich diet with neutral detergent fiber content of 15% by weight did not restore the microbial composition and diversity in the tested animals. Moreover, this perturbation was observed to continue over multiple generations [15,163]. In clinical trials, studies have consistently demonstrated that high-fiber diet intervention, e.g., whole grain cereal, inulin and fructo-oligosaccharide (1:1) mixed fiber, soluble corn fiber, barley kernel-based bread, increases the fecal abundance of several beneficial microbiota, such as *Bifidobacterium* sp. [164,165,166], *Lactobacillus* sp. [166], *Akkermansia* sp. [167,168], *Fecalibacterium* sp. [168], *Roseburia* sp. [168], *Bacteroides* sp. [168,169], and *Prevotella* sp. [170,171]. Moreover, fiber-enriched diets reduce the F/B ratio [169,172] and improve gut microbial diversity [168,170,172]. The relative proportion of Bacteroidetes was reported to be lower in obese people compared with that in lean people [35]. The Bacteroidetes proportion increased on a carbohydrate-restricted low-calorie diet for one year and responded to weight loss [35]. Furthermore, the gut microbiome of obese patients was found to show an increase in beneficial members of *Prevotella*, *Parabacteroides distasonis*, and *Fecalibacterium prausnitzii* after consumption of high-complex carbohydrate diet (low-fat, 28% fat) for one year [173].

Arabinoxylans (AX), arabinoxylan-oligosaccharides (AXOS), and xylo-oligosaccharides (XOS) are commonly found in wheat and are classified as prebiotics, as they specifically increase the pool of beneficial microbiota, including *Bifidobacterium* and *Lactobacillus* [174,175,176,177,178,179,180,181,182]. In a dietary intervention, an AX-enriched diet increased the abundance of *Bifidobacterium* sp. in adults with metabolic syndrome and lowered microbial diversity [174]. AXOS, consisting of arabinoxylooligosaccharides and XOS, can be obtained by enzymatic hydrolysis of AX [183]. AXOS and XOS were shown to increase *Bifidobacterium* sp. and/or *Lactobacillus* sp. [182] in healthy adults [175,176,177,178,179,180] and children [181].

In vitro fermentation of galacto-oligosaccharides (GOS) [184] has been shown to increase *Bifidobacterium* sp. and *Lactobacillus* sp. [185]. A similar observation was reported in a dietary intervention study using GOS on a specific pathogen-free mice model [186]. In clinical trials, GOS at a dose range of 1.5 to 10 g/day when consumed for up to 12 weeks by healthy adults increased the fecal level of *Bifidobacterium* [187,188,189,190,191,192]. A similar dietary intervention in teenage girls (10–13 years old) showed that GOS supplementation at 5 or 10 g/day for three weeks increased *Bifidobacterium* sp. population [193]. In healthy elderly volunteers (65–80 years), administration of GOS at 5.5 g/day for 10 weeks increased *Bifidobacterium* and *Bacteroides* [191]. In addition to *Bifidobacterium* sp., an increase in the relative abundance of lactose-fermenting *Fecalibacterium* and *Lactobacillus* was observed when GOS was consumed by lactose-intolerant volunteers, suggesting that administration of GOS promoted a colonic environment that favors the digestion of lactose [192]. Due to its bifidogenic potential, GOS is included in infant formula to promote a healthy gut microbiome which is dominated by *Bifidobacterium* sp. [184,194,195,196,197,198]. Babies receiving an infant formula containing 4 g GOS/L led to an increase in the abundance of beneficial microbiota *Lactobacillus* and reduced *Clostridium* [184,198]. Lactose-containing baby formula specially formulated with GOS showed significant increases in *Bifidobacterium* and *Lactobacillus* [199]. In a clinical trial for healthy adults, raffinose-oligosaccharide was identified to positively modulate *Bifidobacterium*, and negatively modulate *Clostridium*, viz. *Clostridium histolyticum* and *Clostridium lituseburense* group [200]. In addition, the raffinose-oligosaccharide did not have any significant effect on the diversity of the gut microbiota [200].

Inulin-type fructans (including main dietary sources) have been shown to consistently promote Bifidobacteria. Animal studies have shown that inulin or inulin-type fructan (ITF) could alter gut microbial diversity [201,202], and *Bifidobacterium* sp. was further identified to be promoted by ITF in clinical trials [203,204,205,206,207,208]. Supplementation of ITF at 16 g/day to obese individuals for three months increased the abundance of *Bifidobacterium* [204,207] and *Fecalibacterium* [204], and reduced the abundance of detrimental microbiota of the genus *Bacteroides* [204]. At a lower level of inulin supplementation (10–12 g/day), individuals with mild constipation showed an increase in *Bifidobacterium* [205] or an increase in *Bifidobacterium* and *Fecalibacterium* in healthy adults [203]. In children (mean age 10 years), a similar dose of inulin (10 g/d) for three months only increased the abundance of *Bifidobacterium* but did not affect the level of *Fecalibacterium* or *Bacteroides* [206]. Another study in overweight or obese children (7–12 years, 8 g inulin/day for 16 weeks) showed an increase in *Bifidobacterium* and decrease in *Bacteroides* [208]. Taken together, these studies highlight a range of factors—dosage, inulin type, and other dietary factors (e.g., total fiber intake)—that may contribute to the effectiveness of ITF in modulating key microbes.

Resistant starch is an important substrate for supporting gut health, as it is utilised by a range of beneficial gut microbes [209]. For instance, *Bifidobacterium* sp., *Fecalibacterium* sp., *Eubacterium* sp., and *Ruminococcus* sp. were significantly increased in healthy adults who consumed resistant starch (100 g/day, type 2/ type 4) for three weeks [210]. Individuals with metabolic syndrome demonstrated that resistant starch (type 2), when applied in conjunction with arabinoxylan, could modify gut microbiota towards a beneficial pool (with higher bifidobacterial concentration and less dysbiotic genera), and modified SCFA composition, which resulted in beneficial effects on colonic health and metabolic syndrome [174]. Moreover, a dietary intervention study using resistant starch as a non-digestible carbohydrate confirmed its function to substantially alter the composition of gut microbial species, including *Ruminococcus bromii*, *Eubacterium rectale*, *Collinsella aerofaciens*, and uncultured Oscillibacter group [211].

The intervention of butyrylated high-amylose maize starch was reported in a clinical trial to increase the abundance of the beneficial microbiota, such as *Lactobacillus* sp., *Clostridium coccoides*, *C. leptum* group, and *Ruminococcus bromii*, and reduce the abundance of *R. torques* and *R. gnavus* in the participants who had gut dysbiosis caused by red meat-increased O6-methyl-2-deoxyguanosine adduct level [212].

A six-week dietary intervention study with 10% oligofructose in diet-induced obese rats increased the abundance of *Bifidobacterium* sp., *Lactobacillus* sp., and *Roseburia* sp. and decreased *Clostridium leptum* [213]. In healthy infants, the consumption of infant formula containing oligofructose (3 g/L eight weeks) increased the fecal levels of *Bifidobacterium* sp. [214].

In addition, a synthetic polymer of glucose, polydextrose (PDX) provides similar physiological effects as other dietary fibers and has shown prebiotic potential when tested in animals [215]. Dietary intervention with prebiotics has been shown to selectively stimulate the growth and/or activity of one or a limited number of intestinal bacteria associated with several physiological benefits on health. *Clostridium* clusters I, II, and IV and *Ruminococcus intestinalis* were further reported to be promoted by PDX (8 g/day) in clinical trials for healthy subjects aged 18–50 years during a three-week innervation [216].

Dietary carbohydrates have long been shown to modulate health-beneficial microbes in both humans and animals. A high-fiber diet increased the abundance of *Bifidobacterium* and reduced the ratio of Firmicutes/Bacteroidetes in humans and experimental animals. The prebiotic potential of GOS and other carbohydrates is well known and their supplementation has resulted in an abundance of *Bifidobacterium* sp., *Lactobacillus* sp., *Akkermansia* sp., *Fecalibacterium* sp., *Roseburia* sp., *Bacteroides* sp., and *Prevotella*. Arabinoxylan, resistance starch, and inulin type fructans modulate health-beneficial bacteria, such as *Bifidobacterium, Fecalibacterium,* and *Lactobacillus*. Oligofructose and polydextrose were also found to modulate many health beneficial bacteria, such as *Roseburia, Clostridium lepum,* and *Ruminococcus intestinalis*. Studies have also shown the restorative function of certain carbohydrates on dysbiosis, observed in obese individuals, and hence such carbohydrates could be used as a therapeutic intervention for metabolic diseases.

### 3.2. Fat

A dietary pattern high in saturated and/or total fat is consistently shown to have adverse effects on intestinal microbiome. Fifteen clinical reports (including six randomized controlled interventional studies and nine observational studies) have shown that diets high in total fat and saturated fat have a negative effect on the richness and diversity of gut microbiota [217]. These findings were supported by carefully controlled feeding trials in rodents, which showed that diets containing fat ranging from 44% to 72% increased the F/B ratio of gut microbiota [34,162,218,219,220,221,222,223,224,225,226]. The influences of HFD on modulating microbial abundance, the F/B ratio, and the overall diversity are summarized in Table 4. 

Although it has been indicated that the changes in the F/B ratio in gut microbiota are dependent on the intestinal region and the duration of ingestion [218], changes in the F/B ratio vary with the amount of fat in the diet (Table 4). In rats, the consumption of a mixed HFD (range from 44%−72%) [34,218,219,220,221,222,223,224,225,226] increases the abundance of *Firmicutes* and decreases the proportion of *Bacteroidetes*, thereby leading to an increased ratio of F/B. These bacterial phyla viz. *Bacteroidetes* and *Firmicutes* are commonly known to predominate in the intestinal tract, however, with varying compositions. For example, the genetic obese ob/ob mice displayed fewer *Bacteroidetes* and more *Firmicutes* [34]. The same research team discovered that the obesity phenotype could be transmitted by gut microbiota transplantation in mice. The obesity increased the F/B ratio, and thereby increased the abundance of Firmicutes. After the colonization of “obese-microbiota”, the total body fat in mice significantly increased, and the capacity to harvest energy from the diet was increased as well, thus contributing to the pathophysiology of obesity [227]. However, the amount of dietary fat ranging from 20% to 40% would result in a F/B ratio decrease [228,229,230,231], or no significant changes in their ratio [173,230,232,233]. Furthermore, the gut microbial patterns in HFD-induced rat models showed an abundance of microbes of the order Clostridiales and a decrease in abundance of the microbes of the family Lachnospiraceae, possibly due to its association with body fat percentage [53]. The diminution of *Lactobacillus* population was suggested to be correlated with the high fat and its abundance displayed a negative correlation with body weight and fat mass [162,222,226,229]. In a recent randomized, controlled-feeding clinical trial, 40% fat consumption by healthy young adults was reported to be associated with unfavorable changes in gut microbiota, in that the intervention resulted in an increased abundance of detrimental species from the bacteria *Bacteroides* and *Alistipes*, the two species reported to be abundant in patients with Type 2 diabetes mellitus (T2 DM), and decreased abundance of beneficial bacteria of the genus *Fecalibacterium.* However, 20% of fat consumption showed a positive effect in terms of increasing gut microbiota *Fecalibacterium* sp. and *Blautia* sp. [230]. In people who have metabolic disease, obesity, and coronary heart disease, lowering fat intake to less than 35% fat for two years helped restore the gut microbiome [173,231,233]. The results of this study suggested that low-fat diet intervention for people depends on the degree of metabolic dysfunction, as no change was observed in people if they were not diagnosed with metabolic disease [230]. Furthermore, a sex-dependent effect on shaping the gut microbiota according to diet has been reported recently. Researchers observed a higher abundance of *Roseburia, Holdemania,* and *Desulfovibrio* in men with metabolic syndrome (MetS) than in women with MetS after three years of consumption of the low-fat diet, which led to a detrimental effect in men rather than women [234].

Researchers have also explored the role of saturated and unsaturated fat on the modulation and diversity of gut microbiome. Patterson et al. (2014) [235] showed that different types of dietary fats increased overall gut microbiota diversity but were not significantly different from each other in a mouse model. In a dietary intervention study, the mice fed with palm oil, rich in saturated fatty acid, resulted in a decrease of the Bacteroidetes population, and the changing trends in gut microbiota compositions have been confirmed to be positively correlated with the development of obesity. Similar research found that saturated fat (HFD-containing palm oil) intake (45% fat) induced an elevated F/B ratio in a mice model and has a more stimulatory effect on the development of obesity than mice fed unsaturated fat (olive oil or safflower oil) [236]. The saturated dietary fat altered conditions for gut microbial assemblage by promoting changes in host bile composition, resulting in dysbiosis that can perturb immune homeostasis [228]. In contrast, one of the saturated fats, medium-chain fatty acids, has shown antibacterial effects [237]. Olive oil, high in unsaturated fatty acids, increased commensal bacteria, the populations of *Bacteroidaceae*, in the cecum compared with palm oil, flaxseed oil, and fish oil [235]. Flaxseed/fish oil, when administered together with a low-fat diet, imparted a bifidogenic effect on the host intestinal microbiota composition by increasing the levels of *Bifidobacterium* [235]. *Akkermansia* and *Bifidobacterium* were also considered to be associated with prebiotic consumption and they were reported to show a decreasing trend under the influence of HFD [162,238]. Enteral supplementation with polyunsaturated fatty acids (PUFA) was associated with decreased abundance of detrimental bacteria (e.g., *Streptococcus* sp. and *Escherichia* sp.), greater bacterial diversity in premature infants with an enterostomy [239]. It was also reviewed from the clinical studies that a diet rich in monounsaturated fatty acids decreased total bacterial numbers, whereas a diet rich in polyunsaturated fatty acids had no effect on the richness and diversity of gut microbiota [217].

In summary, both quantity and type of fat in the diet can modulate the F/B ratio and affect both detrimental and beneficial microbes in the gut. In particular, saturated fat consistently lowers health-beneficial microbes, such as *Bifidobacterium* and *Fecalibacterium*, whereas unsaturated fat increases the abundance of *Akkermansia* and *Bifidobacterium* and reduces detrimental bacteria such as *Streptococcus* and *Escherichia* sp. Additionally, saturated fat can increase the F/B ratio and unsaturated fat can lower the F/B ratio, and thereby they could have varying effects on human health depending on the fat quality. Clinical studies to date suggest that a high-fat diet is detrimental to gut health, as it reduces the abundance of beneficial microbes, however this can be reversed if a diet lower in fat is followed.

### 3.3. Protein

Clinical and preclinical studies have suggested that the type and amount of protein in the diet has substantial effects on the gut microbiota (Table 5).

Evidence from animal models suggests that the protein quality affects the composition of gut microbiota. For example, in a preclinical study, it was shown that the cheese whey proteins could act as growth factors for fecal counts of *Lactobacilli* and *Bifidobacteria* compared with Caesin [240]. It was also shown that a whey protein-based diet reduced the abundance of *Clostridium* [241] in the gut. Mung bean protein was shown to reverse the HFD-induced F/B ratio in mice [242]. The mung bean protein also increased the abundance of the family *Ruminococcacea* in an HFD mice model. Based on this observation the authors hypothesized that the bile acid metabolism mediated by *Ruminococcacea* family members would have provided a health benefit in HFD mice [242]. In contrast to the evidence from plant-based protein interventions, diets containing casein increased fecal *Enterobacteriaceae* and decreased fecal *Lactobacilli* in piglets [243]. Furthermore, Bacteroidales and Clostridiales levels were higher in mice when fed a Western diet that contained high levels of meat and seafood [244]. It was also shown that animal-based protein could increase the sensitivity to intestinal inflammation via increasing the potential detrimental gut microbiota (viz. the genera of *Enterococcus*, *Streptococcus*, *Turicibater,* and *Escherichia*, and families Peptostreptococcaceae and Ruminococcaceaea) compared to mice consuming a plant-based protein [245]. A recent clinical study highlighted that casein and soy protein diets should be considered with caution because they appear to disturb normal gene expression in the rectal mucosa of overweight individuals [246]. The authors could not find any changes in microbial diversity or changes in the abundance of specific taxa but could find both beneficial and detrimental metabolites produced by specific microbes. Specifically, amino acid-degrading metabolites were higher, with a reduction in butyrate concentration that the authors found correlated heavily with specific bacteria of the genera *Clostridia, Oscilospira*, *Butyricimonas,* and *Odoribacter* [246].

Although it was suggested that the protein source had a large effect on bacterial community composition [245], other studies have shown that the quantity of proteins is also very important in its effect on gut microbial modulation. In one study [241], mice were fed with a low-fat diet (10% fat) or an HFD (45% fat) for 21 weeks, with either casein (20% kJ) or whey protein isolate (WPI) at 20%, 30%, or 40% kJ. The results of this study showed an increase in abundance of the phylum Proteobacteria and Actinobacteria in the gut microbiota for the experimental animal groups that received 20% WPI. When the protein was increased from 20% to 40% the results were opposite for the phylum Actinobacteria compared to that of the HFD group. A 70-day protein supplementation (a blend of whey isolate (10 g) and beef hydrolysate (10 g)) in a healthy athletes diet had a negative impact on gut microbiota, which resulted in a decreased level of the health-beneficial microbiota, viz. *Roseburia*, *Blautia*, and *Bifidobacterium longum,* and an increase in the microbiota of the phylum Bacteroidetes [247]. The control group, who received maltodextrin, did not show this effect. In addition, different cooking methods of protein could have different effects on gut microbiota. An in vitro study on human gut microbiota showed that the *C.hidtolyticum/perfringens* group, a common food borne pathogen that can produce enterotoxins, causing a wide range of pathologies, was observed in batch fermentations that contained fried meat compared to those containing boiled meat [248], thus suggesting that the cooking method and meat type can influence fermentation profiles within the human gut microbiota.

From the above discussion it is inferred that both the quality and quantity of protein can have effects on the composition and diversity of gut microbiota. Whey protein exerts a bifidogenic effect in HFD mice at a lower concentration and reverses this effect at a higher concentration. Mung bean protein helps to reverse F/B ratio in HFD mice model, and animal-based protein may increase the sensitivity to intestinal inflammation by increasing the potential detrimental gut microbiota. A blend protein supplementation in healthy adults has a negative effect on beneficial microbiota, such as *Roseburia*, *Blautia*, and *Bifidobacterium longum*. Furthermore, it was shown that different cooking methods have different effects on the gut microbiota. More preclinical/clinical trials are required to conclude the effects of various protein supplementation on gut microbiota.

## 4. Summary and Future Perspective

**Figure 1:**
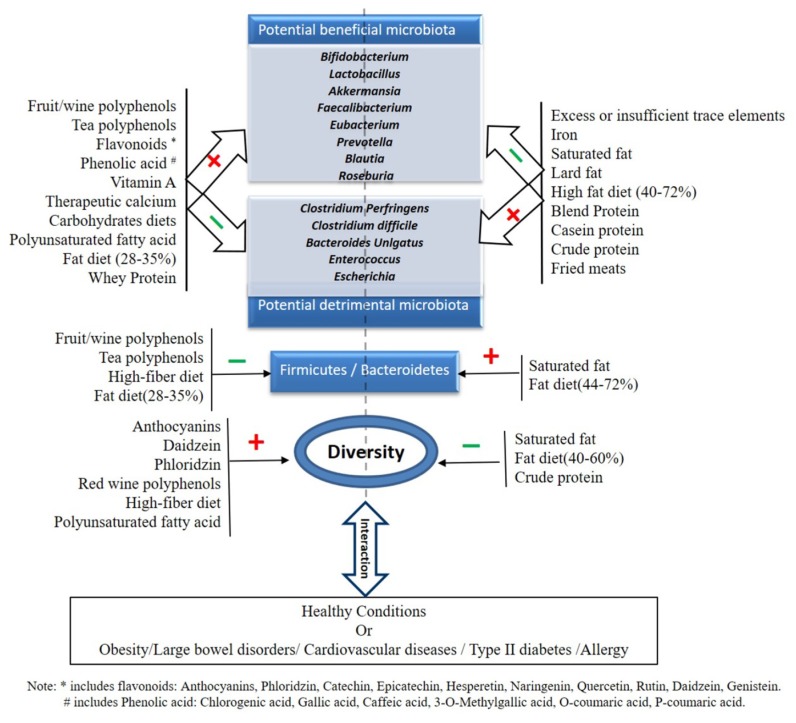
Effect of micro-and macro-nutrients on potential beneficial or detrimental gut microbiota.

Gut dysbiosis is increasingly recognized as a key factor in the development of type 2 diabetes mellitus (T2 DM), cardiovascular disease, childhood allergy/atopy, and many other metabolic and infectious diseases [249,250]. The gut microbiota is a potential target to improve human health [251,252], and dietary components (both micro- and macro-nutrients) are recognized as playing an important role (Figure 1).

There is consistent evidence suggesting that polyphenols have considerable effects on promoting the abundance of beneficial microbes in the gut, which may contribute to favorable health outcome beyond the gut. Specific polyphenols in animal models have been shown to reduce the F/B ratio. In vitro assays have also shown the ability of specific polyphenols to reduce the abundance of detrimental/pathogenic microbes. The major beneficial microbes positively modulated with polyphenols include *Bifidobacterium, Lactobacillus, Akkermansia,* and *Fecalibacterium* sp. Some specific polyphenols modulate other beneficial microbes, such as *Clostridium coccoides-Eubacterium rectale* group, *Eubacterium dolichum, Lactococcus lactis, Ruminococcus torques, Clostridium hathewayi, Bacteroides uniformis, Prevotella sp., Blautia coccoides,* and *Eggerthella lenta* (Table 1 and Figure 1). Vitamins form the second major micronutrient that modulates the health-beneficial gut microbes. Vitamins show a distinct response on modulating health beneficial/detrimental microbes and vitamin A, C, D, and E supplementation has a positive influence on health-beneficial microbes, such as *Bifidobacteria, Akkermansia,* and *Lactobacilli*. The restorative function of normal flora in the dysbiotic gut by vitamin A in ASD patients promises to explore therapeutic potential of vitamin A in ASD and other similar diseases. Vitamin B administration should be cautioned, as it can activate virulence of pathogenic organisms and can also positively modulate some detrimental bacteria. There is an acute scarcity in the literature that explores the role of minerals and trace elements in modulating gut microbiota. However, supplementation of calcium, magnesium, phosphorus, selenium, and zinc has shown that there may be some small effects that support beneficial gut bacteria, which include *Akkermansia, Bifidobacterium,* and *Ruminococcus*. Iron supplementation in iron-deficient infants has been shown to modulate detrimental and potentially pathogenic microbiota. Iodine supplementation leads to a decrease in the abundance of *Fecalibacterium prausnizii* and selenium supplementation leads to a decrease in the abundance of *Dorea* and *Mucisprillum*. More studies are needed to conclude these effects.

Under macronutrients, carbohydrates form the major modulator for health-beneficial microbes. Dietary fiber, arabinoxylan, GOS, Inulin type fructan, resistant starch, and polydextran have major bifidogenic effects and can positively modulate health-beneficial microbes in the gut. The major health-beneficial microbes modulated by these major carbohydrates are *Bifidobacterium sp., Lactobacillus sp, Akkermansia sp, Fecalibacterium sp., Roseburia sp., Bacteroides sp. and Prevotella, Roseburia, Clostridium lepum* and *Ruminococcus intestinalis* (Table 4 and Figure 1). Specific carbohydrates are also found to reduce the F/B ratio. Animal experimentation has shown that saturated and unsaturated fats have opposing effects on the modulation of gut microbiota. Compared to saturated fats, diets containing unsaturated fat increase the abundance of beneficial gut microbiota and reduce detrimental gut microbiota (Table 4 and Figure 1). A high-fat diet could lead to gut dysbiosis and thereby increase the F/B ratio. A Mediterranean diet and a low-fat high carbohydrate diet modulated two butyrate producers (*Roseburia* sp. and *Fecalibacterium prausnitzii*) in the gut of an obese population and was associated with insulin sensitivity in these populations [231]. This study further confirmed the health benefits of a low-fat diet via the modulation of gut microbiota. High intake of dietary protein lowers the abundance of microbes that have been associated with beneficial health effects and it is not clear whether this differs between proteins from animal- or plant-based sources. The quality and quantity of the dietary protein influences the gut microbiota. There is an acute scarcity of studies using defined proteins that explore their action on gut microbial modulation. More clinical/preclinical studies are required to make an informed decision on protein supplementation for modulating health-beneficial microbes.

It is further clear in the current review that macro- and micro-nutrients greatly influence the composition and/or diversity of the gut microbiome. For example, fermentable dietary fibers, which include AX, RS, inulin, oligosaccharides, and GOS, increase the abundance of *Bifidobacterium*, *Lactobacillus*, *Roseburia*, *Bacteroides*, *Akkermansia*, butyrate-producing *Fecalibacterium,* and *Ruminococcus* at the genus level, which are associated with various health benefits, whereas diets containing more than 44% energy from fat increase the ratio of F/B, which can be attenuated by red wine/tea-derived polyphenols. High intakes of dietary protein lower the abundance of microbes that have been associated with beneficial health effects and it is not clear whether this differs between proteins from animal- or plant-based sources. Moreover, the role of some micronutrients (e.g., Vitamin D and Calcium) in modulating gut microbiota towards a healthy phenotype by increasing *Bifidobacterium*, *Lactobacillus*, restoring *Ruminococcus* and *Akkermansia* in obese models has indicated that a favorable health outcome could be mediated by dietary intervention studies. 

Although this current review summarized key current research outcomes on the effect of specific micro and macro nutrients on modulating gut microbiota, there is a lack of data on how these specific nutrient components alter the gut microbiota in humans. Humanized mouse models and gnotobiotic mouse models could provide further information. Although these models have several limitations, they enable dietary patterns and nutrients to be carefully controlled and microbial changes to be evaluated. Additionally, well-defined dietary intervention studies are needed that utilize a diverse range of individuals to better understand the intra- and inter-individual variability in how individuals and their microbiomes respond differently to dietary patterns and specific food component.

## Figures and Tables

**Table 1:** Modulations of dietary polyphenols on potentially beneficial and detrimental gut microbiota.

	Dose and Treatment Duration/Test Model	F/B	Potentially Beneficial Microbiota													Potentially Detrimental Microbiota					Species	Diversity
			*Bifidobacterium* sp.	*Lactobacillus* sp.	*Akkermansia* sp.	*Fecalibacterium* sp.	*Eubacterium* sp.	*Bacteroides* sp.	*Prevotella* sp.	*Blautia* sp.	*Clostridium* sp.	*Ruminococcus* sp.	*Eggerthella* sp.	*Roseburia* sp.	*Coprococcus* sp.	*Ruminococcus* sp.	*Clostridium* sp.	*Bacteroides* sp.	*Enterobacter* sp.	*Enterococcus* sp.		
Anthocyanins																						
Marques et al. (2018)	Standard diet with Blackberry anthocyanin rich extract (25 mg/Kg body weight per day) for 17 weeks and standard diet as reference/rat model [57]																					↑
	High-fat diet (HFD) with blackberry anthocyanin rich extract (25 mg/Kg body weight per day) for 17 weeks and HFD as reference/rat model [57]				↑											↓						↑
Catechin																						
Tzounis et al. (2010)	A high-cocoa flavanol group (21 mg catechin/day) for four weeks of treatment and a low-cocoa flavanol group (3 mg catechin/day) as reference/clinical trials (22 healthy human volunteers) [63]		↑	↑													↓					
Tzounis et al. (2008)	Catechin (150 mg/L) inoculated in stirring batch-culture vessels containing fecal slurry (1:10, w/v), for treatment up to 48 h and incubating flavanol monomers in medium without fecal slurry inoculation as reference/in vitro [48]		↑								↑						↓				*Clostridium coccoides–Eubacterium rectale* group; *C. histolyticum* group	
Caffeic acid																						
Parkar et al. (2013)	Caffeic acid (10 μg/mL) in fermentation medium for 48 h and a control without polyphenol as reference/in vitro [49]	↑	↑					↑														
Chlorogenic acid																						
Parkar et al. (2013)	Chlorogenic acid (30 and 100 μg/mL) in fermentation medium for 48 h and a control without polyphenol as reference/in vitro [49]	↑	↑					↓														
Daidzein																						
Iino et al. (2019)	Diadzin intake (three traditional Japanese soybean products: natto, tofu, and fried tofu) was calculated based on brief self-administered diet history questionnaire/clinical trials (1044 healthy subjects: 411 men and 633 women) [68]																					↑
Epicatechin																						
Tzounis et al. (2008)	Epicatechin (1000 mg/L) inoculated in stirring batch-culture vessels containing fecal slurry (1:10, w/v), for treatment up to 48 h and incubating flavanol monomers in medium without fecal slurry inoculation as reference/in vitro [48]						↑				↑										*E. rectale;* *C.coccoide*	
Genistein																						
Paul et al. (2017)	Genistein diet (0.25 g/kg) for 4 weeks and the control group without genistein as reference/mice model (humanized germ-free mice that received fecal transplantation from breast cancer patients) [62]			↑	↑		↑				↑	↑						↓			*A. municiphila* *Ruminococcus torques* *Clostridium hatheway* *Bacteroides eggerthii Bacteroides ovatus* *Eubacterium dolichum*	
Phloridzin																						
Mei et al. (2016)	Phloridzin (20 mg/kg/day) for 10 weeks and the control (vehicle treated) as reference/mice model (type 2 diabetes mice model) [58]				↑																*A. muciniphila*	↑
Quercetin																						
Etxeberria et al. (2015)	Quercetin supplementation (30 mg/kg body weight/day) for 6 weeks and the control group as reference/rat model (rats fed HFD) [41]	↓																				
Epigallocatechin-3-gallate and resveratrol (EGCG + RES)																						
Most et al. (2017)	EGCG + RES group (282 mg/d, 80 mg/day) for 12 weeks and baseline as reference/clinical trials (19 subjects: overweight, obese females, 37.8 ± 1.6 years, BMI 29.6 ± 0.5 kg/m^2^) [72]					↓		↓													*F. prausnitizii*	
Fruit-derived polyphenols																						
Mayta-Apaza et al. (2018)	Tart cherry juice consumption (8 oz/day) for 5 days and baseline as reference/clinical trials (10 healthy participants with low-bacteroides: 5 males, 5 females, aged 23–30 years) [70]		↑					↑	↑							↓						
	Tart cherry juice consumption (8 oz/day) for 5 days and baseline as reference/clinical trials (10 healthy participants with high-bacteroides: 5males, 5females, aged 23–30 years) [70]		↓					↓								↑						
Henning et al. (2017)	Vegetable/fruit juice-based juices (consumed 6 bottles, 16 oz./bottle) for 3 days and baseline as reference/clinical trials (25 healthy subjects: 18–50 years of age with a custom diet including < 3 servings of fruits/vegetables per day) [66]							↑														No significant effect
Li et al. (2015)	Pomegranate (POM) extract (1000 m/d, total phenolic content expressed as gallic acid equivalents of 680 μg/g) for 4 weeks and baseline as reference/clinical trials (20 healthy adults: 9 women and 11 men) [71]			↑	↑				↑												*A. muciniphila*	
Chacar et al. (2018)	Different concentrations of grape phenolic compounds (2.5 and 5 mg/kg/d diluted in 0.1% Dimethyl Sulfoxide) for 14 months and the control group (0.1% Dimethyl Sulfoxide alone) as reference/rat model (30, 2-mo-old rats) [59]		↑																			
Collins et al. (2016)	A diet of high fat (HF) with an equal combination of the extractable and non-extractable grape-derived polyphenols (EP and NEP) for 16 weeks and the HF diet group as reference/mice model [53]											↑										No significant effect
Taira et al. (2015)	A diet of high fat (HF) with polyphenols from aronia, haskap, and bilerry separately (anthocyanin content was 0.4%) for 4 weeks and the HF diet group as reference/rat model [42]	↓						↓														
Neyrinck et al. (2013)	A diet of high fat (HF) with pomegranate peel-derived polyphenols (6 mg/d per mouse) for 4 weeks and the HF diet group as reference/mice model [54]		↑																			
Viveros et al. (2011)	Antibiotic-free diets containing grape pomace concentrate (60 g/kg) or grape seed extract (7.2 g/kg) for 21 days and the antibiotic-free diets group as reference/broiler chicks model [60]			↑							↑						↓			↑		↑
Mayta-Apaza et al. (2017)	Microbial suspensions (25 mL) of human distal colon compartments cultured with apricots/cherries (5 mL or g), 37 °C for 48 h and stool samples with a low diversity microbiota (dominated by Verrumicrobia and Synergistes) as reference/in vitro (68 human stool samples) [69]							↑														
Red wine-derived polyphenols																						
Moreno-Indias et al. (2016)	Participants drank red wine (272 mL/d) for 30 days and baseline as reference/clinical trials (metabolic syndrome patients) [64]	↓	↑	↑		↑			↑					↑					↓		*F. prausnitzii* *E. cloacae*	↑
Queipo-Ortuño et al. (2012)	Red wine polyphenol (272 mL per day, containing polyphenol of 733–797 mg) for 4 weeks and the baseline as reference/clinical trial (healthy men) [65]		↑				↑	↑	↑	↑			↑							↑	*Bacteroides uniformis* *Eggerthella lenta* *Blautia coccoides Eubacterium rectale*	↑
Dolara et al. (2005)	Red wine polyphenols (50 mg/kg) for 16 weeks and the HFD group as reference/rat model [55]		↑	↑													↓					No significant effect
Tea-derived polyphenols																						
Jin et al. (2012)	Green tea (1000 mL/day) for 10 days and baseline as reference/clinical trials [67]		↑																			
Wang et al. (2018)	TP-HFD groups (TP Low- HFD group with 0.05% TP, TP Middle- HFD group with 0.25% TP, TP High- HFD group with 0.8% TP) for 8 weeks and HFD group as reference/mice model [43]	↓																				↑
Seo et al. (2015)	HFD with fermented green tea group for 8 weeks and HFD group as reference/mice model [44]	↓																				

Note: (↑): increased F/B or microbiota population; (↓): reduced F/B or microbiota population; Blank: not reported. The potentially beneficial microbiota and potentially detrimental microbiota listed in the form were based on the information from the literature of the reviewed studies, excluding the taxa without clear description of their functions.

**Table 2:** Modulations of dietary vitamins, minerals, and trace elements on potentially beneficial and detrimental gut microbiota.

	Does and Treatment Duration/Test Model	F/B	Potentially Beneficial Microbiota											Potentially Detrimental Microbiota												Species	Diversity
			*Bifidobacterium* sp.	*Lactobacillus* sp.	*Akkermansia* sp.	*Ruminococcus* sp.	*Fecalibacterium* sp.	*Dialister* sp.	*Allobaculum* sp.	*Blautia* sp.	*Clostridium* sp.	*Roseburia* sp.	*Lactococcus* sp.	*Enterobacteria* sp.	*Escherichia* sp.	*Salmonella* sp.	*Bacteroides* sp.	*Ruminococcus* sp.	*Veillonella* sp.	*Clostridium* sp.	*Coprococcus* sp.	*Shigella* sp.	*Oscillibacter* sp.	*Enterococcus* sp.	*Pseudomonas* sp.		
Vitamin A																											
Huda et al. (2019)	One dose of 50,000 IU vitamin A within 48 h of birth and placebo as reference/clinical trial (306 infants in early (6–15 week) or late (2 y) infancy) [81]		↑		↑																						
Liu et al. (2017)	A dose of 200,000 IU vitamin A once orally for a 6-month follow-up intervention and baseline as reference/cinical trial (64 children with autism spectrum disorder, aged 1 to 8 years old) [79]		↓														↑										
Lv et al. (2016)	Vitamin A—deficient and vitamin A—normal as reference/clinical trial (59 pediatric patients with persistent diarrhea aged 1–12 months) [80]										↓													↑		*Clostridium butyricum*	↓
Lee and Ko (2017)	1 mg/kg/day of retinoic acid, the metabolite of dietary vitamin A on murine norovirus infection mice for 8 days and the murine norovirus infection mice as reference/mice model [76]		↑	↑				↑	↑																		
Lee et al. (2016)	1 mg/kg/day of retinoic acid, the metabolite of dietary vitamin A for 8 days and the control group as reference/mice model [75]		↑					↑	↑																		
	1 mg/kg/day of retinoic acid, the metabolite of dietary vitamin A on murine norovirus infection mice for 8 days, and the murine norovirus infection mice as reference/mice model [75]			↑																							
Vitamin B																											
Miki et al. (2017)	Oral gavage with pyridoxine hydrochloride (vitamin B6) at 100 μg/mouse (Sigma) at 1–7 days after infection and baseline as reference/mice model (streptomycin-treated and *Salmonella enterica* serovar Typhimuirum-infected mice) [88]															↓											
Vitamin C																											
Li et al. (2017)	Mean 294.9 mg/d for men and mean 189.8 mg/day for women (3-day food dairy using household measures) and baseline as reference/clinical trial (16 free-living adults with cystic fibrosis) [90]																↓										
Vitamin D																											
Naderpoor et al. (2019)	> 75 nmol/L 25-hydroxyvitamin D (25(OH)D) and vitamin D-deficient (≤ 50 nmol/L) as reference/clinical trial (25 overweight or obese (BMI ≥25 kg/m^2^) healthy adults) [111]					↓												↓			↑						No effect
Garg et al. (2018)	A dose of 40,000 IU vitamin D once weekly for 8 weeks using two capsules of 20,000 IU (Plenachol, Encap) and baseline as reference/clinical trial (patients with vitamin D deficiency: 25[OH]D < 50 nmol/L) [107]													↑													No effect
Talsness et al. (2017)	Maternal supplementation of vitamin D was carried out at 0μg/day, <10 μg/day, or ≥ 10 μg/day during pregnancy and the fecal samples from their one-month old babies were tested for gut microbiota/clinical trial [109]		↓								↓						↑			↓						*Bacteroides fragilis;* *Clostridium difficile*	
Kanhere et al. (2017)	50,000 IU of oral vitamin D3 supplemented for 12 weeks and placebo as reference/clinical trial (Cystic Fibrosis patients as model) [98]												↑						↓								
Luthold et al. (2017)	vitamin D intake ≥ 10 μg/day in three tertiles (tertile 1: 4.21–18.93 ng/mL, tertile 2: 18.93–26.48 ng/mL, tertile 3: 26.48–61.30 ng/mL) and the highest vitamin D intake as reference/clinical trial (150 young healthy adults) [110]		↑																		↑						
Bashir et al. (2016)	Vitamin D3 supplementation with weekly dose of 980 IU/kg bodyweight for 4 weeks, and 490 IU/kg bodyweight for the remaining 4 weeks, the baseline as reference/clinical trial (healthy volunteer) [103]														↓										↓		↑
Ciubotaru et al. (2015)	Vitamin D supplementation with weekly ergocalciferol (50,000 IU) on stable normal glucose tolerance for 12 months and the same treatment on stable prediabetes as reference/clinical trial [97]	↓				↓	↓	↓																			
Vitamin E																											
Tang et al. (2016)	Vitamin E (18 mg/day) added iron therapy (6 mg/kg/d) for 8 weeks and placebo added iron therapy (6 mg/kg/day) as reference/clinical trial (infants and toddlers who were at risk of iron deficiency) [119]											↑															
Choi et al. (2019)	High vitamin E group (0.18 mg/20 g of bw per day) treated by oral gavage for 34 days, the control group (0.2 mL of corn oil) and low vitamin E group (0.06 mg/20 g of bw per day) as reference/mice model [117]	↓																									
Calcium																											
Trautvetter et al. (2018)	Supplementation of 1000 mg calcium +1000 mg phosphorus/day for 8 weeks and the supplementation of 1000 mg phosphorus/day as reference/clinical trials (healthy men) [124]										↑															*Clostridium* XVIII	
	Supplementation of 1000 mg calcium +1000 mg phosphorus/day for 8 weeks and the supplementation of 500 mg calcium + 1000 mg phosphorus/day as reference/clinical trials (healthy men) [124]		↓								↑															*Clostridium* XVIII	
Li et al. (2018)	Maternal insufficient calcium intake (2.5 g/kg) / Maternal excess calcium intake (12 g/kg) for 8 weeks will influence the gut microbiota in the offspring/mice model [126]	↑*																									
Chaplin et al. (2016)	HFD enriched with calcium supplementation (12 g/kg) for 54 days and HFD group (4 g/kg) as reference/mice model [127]		↑	↑																							
Borda-Molina et al. (2016)	Diet mixed with the supplementation of calcium (3 g/kg) for 10 days and diet without calcium group as reference/broiler chickens model [151]																										↓
Aslam et al. (2016)	HFD enriched with calcium supplementation (5.25 g/kg) for 18 months and HFD group (0.41 g/kg) as reference/mice model [125]				↑	↑																					↑
Magnesium^**^																											
Jørgensen et al. (2015)	Dietary magnesium deficiency (0.02% magnesium) for 6 weeks and standard diet (0.2% magnesium) group as reference/mice model [130]																										↓
Winther et al. (2015)	Dietary magnesium deficiency (0.02% magnesium) for 6 weeks and standard diet (0.2% magnesium) group as reference / Mice model [131]																										No significant effect
Pachikian et al. (2010)	Magnesium-deficient diet (70 mg/kg) for 4 days and control diet (500 mg/kg) group as reference/mice model [129]		↓																								
	Magnesium-deficient diet (70 mg/kg) for 21 days and control diet (500 mg/kg) group as reference/mice model [129]		↑	↑																							
Iron																											
Kotryna Simonyté Sjodin et al. (2019)	High-iron-fortified formula (6.4 mg Fe/day) for 45 days and the baseline as reference/clinical trials (6-month-old healthy Swedish infants) [139]		↓																								
	High-iron-fortified formula (6.4 mg Fe/day) for 45 days and low-iron-fortified formula (1.2 mg Fe/day) as reference/clinical trials (6-month-old healthy Swedish infants) [139]		↓	↑																							
	High-iron-fortified formula (6.4 mg Fe/day) for 45 days and iron drops (no-added-iron formula with liquid ferrous sulfate supplementation (5.7 mg Fe/day) as reference/clinical trials (6-month-old healthy Swedish infants) [139]			↑													↓										
Tang et al. (2017)	Multiple micronutrient powder containing 12.5 mg iron daily for 3 months and multiple micronutrient powder without the iron as reference/clinical trials (6-month-old Kenyan infants) [135]		↓												↑					↑							
Lee et al. (2017)	Iron therapy via Per Oral over 3 months and via Intravenous as reference/clinical trials (IBD patients with anemia) [149]					↓	↓																			*Fecalibacterium prausnitzii;* *Ruminococcus bromii*	
Jaeggi et al. (2015)	Home-fortified maize porridge containing 2.5 mg iron daily for 4 months and porridge without the iron as reference/clinical trials (6-month-old Kenyan infants) [134]											↑			↑							↑				*Escherichia coli*	
Jaeggi et al. (2015)	Home-fortified maize porridge containing 12.5 mg iron daily for 4 months and porridge without the iron as reference/clinical trials (6-month-old Kenyan infants) [134]		↓												↑		↑			↑		↑				*Escherichia coli*	
Dostal et al. (2014)	Oral tablets containing 50 mh Fe for 4 d/week, last for 38 weeks, and the placebo as reference/clinical trial (6–11 rural South African children with Fe deficiency) [138]																										
Zimmermann et al. (2010)	Iron-fortified biscuits containing 20 mg Fe/d, and 4 times/week, last for 6 months, and the nonfortified biscuits as reference/clinical trial (6–14 Ivorian children) [136]			↓										↑		↑											↑
Fang et al. (2018)	1 mL liquid iron (Liquid iron preparations were prepared by dissolving FeSO_4_ salt in 1 mL of 0.01 mol/L HCL) preparation containing 8 mg, 16 mg, or 24 mg of iron for 30 days and control group (0.01 mol/L HCI) as reference/rat model [140]								↓												↑						↑
Constante et al. (2017)	Iron-sufficient diet (50 mg/kg) for 4 weeks and iron-deficient diet (5 mg/kg) as reference/mice model [145]												↓				↑							↑		*Bacteroides caccae*	
	Iron-supplemented diet (500 mg/kg) for 4 weeks and iron-sufficient diet (50 mg/kg) as reference/mice model [145]																							↑			
	Ferrous bisglycinate (FBG) (50 mg/kg) for 4 weeks and ferrous sulfate (FS) (50 mg/kg) as reference/mice model [145]																										
	Ferric ethylenediaminetetraacetic acid (FEDTA) for 4 weeks and ferrous sulfate (FS) (50 mg/kg) as reference/mice model [145]											↓	↓								↓						
Kortman et al. (2016)	Medium with iron (50 or 250 µmol/l ferrous sulfate, 50 or 250 µmol/l ferric citrate, or 50 umol/L hemin) incubated for 44 h at 37 ℃ and medium without supplementary iron as reference/in vitro (Human fecal sample) [141]																										
Phosphorus																											
Borda-Molina et al. (2016)	Diet mixed with the supplementation of Phosphorus (3 g/kg) for 10 days and diet without calcium group as reference/broiler chickens model [151]			↑↓#			↑																			*Lactobacillus taiwanensis*	↑↓
Witzig et al. (2015)	Diet mixed with the supplementation of phosphorus for 10 days and diet without phosphorus group as reference/broiler chickens model [253]			↑ ↓#																						*Lactobacillus taiwanensis;* *Lactobacillus salivarius;* *Lactobacillus crispatus;* *Lactobacillus reuteri*	↑↓
Zinc																											
Zackular et al. (2016)	High Zn diet (1000 mg/kg) for 5 weeks and control diet (29 mg/kg) as reference/mice model [155]																			↑				↑		*Clostridium* XI	↓
Reed et al. (2015)	Zn diet (42 ug/g) for 28 days and Zn deficiency diet (2.5 ug/g) as reference/chicks model [153]													↓										↓			↑
Shao et al. (2014)	Supplemental Zn (120 mg/kg) diet for 42 days and diet without Zn as reference/Salmonella Typhimurium-challenged Broiler chicken model [154]			↑												↓											↑
Selenium																											
Zhai et al. (2018)	Selenium diet (added amounts of Se 0.4 mg/kg) for 8 weeks and Se-deficient diet (Se level < 0.01 mg /kg) as reference/mice model [158]				↑																						Not significant effect
Kasaikina et al. (2011)	Selenium diet (added amounts of Se 0, 0.1, 0.4, and 2.25 ppm) for 10 weeks and Se-deficient diet (0 ppm Se diet) as reference/mice model [157]																										↑
Iodine																											
Shen et al. (2019)	18μg/kg/d iodine for 8 weeks and un-treated obese mice as reference/HFD-induced obesity mice model [159]								↑	↓										↑			↑	↑			
	18μg/kg/d iodine for 8 weeks and control group as reference/mice model [159]		↑	↑			↑		↑	↓		↑														*Fecalibacterium prausnizii*	

* Insufficient calcium for mother mice leads to change in their offspring; ** Magnesium deficient; ^#^ Changes in different sections of gastrointestinal tract of broiler chickens. Note: (↑): increased F/B or microbiota population; (↓): reduced F/B or microbiota population; Blank: not reported. The potentially beneficial microbiota and potentially detrimental microbiota listed in the form were based on the information from the literature of the reviewed studies, excluding the taxa without a clear description of their functions.

**Table 3:** Modulations of dietary carbohydrates on potentially beneficial and detrimental gut microbiota.

	Dose and Treatment Duration/Test Model	F/B	Potentially Beneficial Microbiota											Potentially Detrimental Microbiota				Species	Diversity
			*Bifidobacterium* sp.	*Lactobacillus* sp.	*Akkermansia* sp.	*Fecalibacterium* sp.	*Roseburia* sp.	*Eubacterium* sp.	*Bacteroides* sp.	*Prevotella* sp.	*Clostridium* sp.	*Ruminococcus* sp.	*Eggerthella* sp.	*Ruminococcus* sp.	*Clostridium* sp.	*Bacteroides* sp.	*Enterococcus* sp.		
Arabinoxylan																			
Hald et al. (2016)	Arabinoxylan (AX)-enriched diet (whole-grain rye and enzyme-treated wheat bran) for 4 weeks and baseline as reference/clinical trials (24 subjects: 39–75 years with metabolic syndrome) [174]		↑																↓
	Arabinoxylan (AX)-enriched diet (whole-grain rye and enzyme-treated wheat bran) for 4 weeks and a low-fiber western-style diet (refined grains and a minimal concentration dietary fiber) as reference/clinical trials (24 subjects: 39–75 years with metabolic syndrome) [174]		↑																↓
Arabinoxylan-oligosaccharides																			
Windey et al. (2015)	Wheat bran extract (10 g/day) containing arabinoxylan-oligosaccharides for 3 weeks and placebo (maltodextrin, 10 g/day) as reference/clinical trials (20 healthy subjects: 17 women and 3 men; age range 19–44 years; BMI range 18.7–24.3 kg/m^2^) [182]			↑														*Bifidobacterium adolescentis*	
François et al. (2014)	WBE (enriched AXOS) at 5 g/day for 3 weeks and placebo as reference/clinical trials (29 healthy children, age range of 8–12 years) [181]		↑																
Walton et al. (2012)	AXOS treatment (2.2 g) for 21 days and placebo as reference/clinical trials (40 healthy adults: 20 males and 20 females, mean age 31.4 years (± 8.9), average BMI 23.3 kg/m^2^ (± 2.8)) [178]		↑																
François et al. (2012)	Wheat bran extract (WBE, 10 g/day)-enriched AXOS for 3 weeks; both WBE (3 g/day) group and placebo group as references/clinical trials (63 healthy adults) [176]		↑																
Cloetens et al. (2010)	Arabinoxylan-oligosaccharides (AXOS, 10 g/d) for 3 weeks and placebo as reference/clinical trials (20 healthy subjects: 14 women, 6 men; mean age 24 (sd 5) years, mean BMI 20.9 (sd 2.3) kg/m^2^) [175]		↑																
Xylo-oligosaccharide																			
Childs et al. (2014)	XOS (8 g/day) for 21 days and placebo as reference/clinical trials (44 healthy adults, 25–65 years) [179]		↑																
Finegold et al. (2014)	XOS (1.4 g/day or 2.8 g/day) for 8 weeks and the placebo as reference/clinical trials (32 healthy adults) [180]		↑																
Lecerf et al. (2012)	Xylo-oligosaccharide (XOS, 5 g/d) for 4 weeks and placebo (wheat maltodextrin, 5 g/day) as reference/clinical trials (60 healthy volunteers) [177]		↑																
Galacto-oligosaccharides																			
Azcarate-Peril et al. (2017)	GOS ( > 95% purity) treatment in 5 d increments according to a fixed schedule from 1.5 g/day to 15 g/day up to 36 days and placebo (Sweetose) as reference/clinical trials (52 lactose-intolerant individuals, mean age of 41 year, mean BMI of 27.1) [192]		↑	↑		↑									↓				
Civardi et al. (2017)	GOS (7 g/L) for 60 ± 5 days and standard formula without GOS as reference/clinical trials (117 healthy infants: formula milk supplemented with functional ingredients (*n* = 55), or a standard formula (*n* = 62)) [197]		↑																
Paganini et al. (2017)	Iron-containing micronutrient powders (MNPs) with GOS (7.5 g/day) for 4 months; MNPs without iron and MNPs with 5 mg iron as references/clinical trials (Kenyan infants aged 6.5–9.5 months (*n* = 155)) [198]		↑	↑											↓				
Musilova et al. (2015)	GOS (9 g/day) and maltodextrins (1 g/d) for 5 days and baseline as reference/clinical trials (fecal samples of 11 healthy adults: 6 women and 5 men; mean age, 35.18 ± 10.91 years) [190]		↑																
Vulevic et al. (2015)	GOS (5.5 g/day) for 10 weeks and placebo (maltodextrin) as reference/clinical trials (40 elderly volunteers: 25 women and 15 men; range age of 65–80 years) [191]		↑						↑										
Sierra et al. (2015)	GOS (0.44 g/day) for 6 months with additional feeding of GOS (0.5 g/dl) for 6 months and placebo as reference/clinical trials (365 healthy infants had a gestational age of 37–42 weeks and a birth weight greater than 2500 g) [196]		↑																
Giovannini et al. (2014)	GOS-supplemented formula (0.4 g/100 mL) for up to 70 days and the identical formula without GOS as reference/clinical trials (160 healthy infants: gestational age from 37 to 42 completed weeks; birth weight ≥ 2500 g) [184]		↑	↑											↓				
Whisner et al. (2013)	Smoothie drinks with daily GOS intake 0 g, 5 g, and 10 g for three 3-week periods in a random order and baseline as reference/clinical trials (31 healthy adolescent girls: age 10–13 years) [193]		↑																
Westerbeek et al. (2013)	Mixture of neutral and acidic oligosaccharides (scGOS/lcFOS/pAOS) in increasing doses between days 3 and 30 of life to 1.5 g/kg/day, and the placebo as reference/clinical trials (113 healthy infants) [195]		↑																↓
Scalabrin et al. (2012)	Formula with polydextrose (PDX) and galacto-oligosaccharides (GOS) (4 g/L, 1:1 ratio) for 60 days and control formula (Enfamil Lipil) as reference/clinical trials (230 healthy infants: 21- to 30-days-old with 37 to 42 weeks of gestational age, birth weight ≥2500 g) [194]		↑																
Walton et al. (2012 b)	GOS (4 g) twice/d for 3 weeks and placebo as reference/clinical trials (39 volunteers: age 50–81 years, BMI of 19.7–38.4 kg/m^2^) [189]		↑																
Davis et al. (2011)	GOS with four dosages (0, 2.5, 5, and 10 g/d) for 3 eewks each (12 weeks in total) and baseline as reference/clinical trials (18 healthy volunteers) [188]		↑																
Davis et al. (2010)	GOS-containing chocolate chews with dosage levels (5.0 g and 10.0 g) for 3 weeks and baseline as reference/clinical trials (18 subjects: 13 males and 5 females, ages of 19 to 50 years old) [187]		↑																
Monteagudo-Mera et al. (2016)	GOS (90% purity, 40 mL/day equivalent of 0.26 g/kg bodyweight of GOS) for 14 days and baseline as reference/mice model (four pathogen-free mice) [186]		↑	↑					↑		↑				↓				
Ladirat et al. (2014)	GOS (4.2 mg/mL) and 4 antibiotics (1 or 10 μg/mL) in the medium for healthy adult fecal sample fermentation for up to 48 h and antibiotic-treated samples as reference/in vitro (8 healthy adult fecal samples) [185]		↑	↑															
Raffinose-oligosaccharide																			
Fernando et al. (2010)	Diet fortified with canned chickpeas (200 g/day) or raffinose oligosaccharide (5 g/day) for 3 weeks and control diet as reference/clinical trials (12 healthy adults: 18–65 years) [200]		↑												↓			*Clostridium* clusters I/II and XI; *Clostridium histolyticum; Clostridium lituseburense* group	No significant effect
Lactose																			
Francavilla et al. (2012)	Formula with no lactose for 2 months followed by an identical lactose-containing (3.8% lactose) formula for an additional 2 months to infants with cow’s milk protein allergy (CMA), and the formula with no-lactose as reference/clinical trials (28 infants with CMA) [199]		↑	↑															
Inulin/inulin-type fructans																			
Drabinska et al. (2018)	Oligofructose-enriched inulin (Synergy 1) (10 g/d) for 3 months and placebo (maltodextrin; 7 g/day) as reference/clinical trials (34 pediatric celiac disease patients, mean age 10 years, 62% females, on a gluten-free diet) [206]		↑																
Vandeputte et al. (2017)	Chicory-derived Orafti inulin (12 g/day) for 4 weeks and placebo as reference/clinical trials (42 healthy adults with mild constipation) [205]		↑																No significant effect
Nicolucci et al. (2017)	Oligofructose-enriched inulin (8 g/day) for 16 wks and placebo (maltodextrin) as reference/clinical trials (42 healthy children, 7–12 years, overweight or obese >85 th percentile of body mass index) [208]		↑													↓		*Bacteroides vulgatus*	
Salazar et al. (2014)	ITF (16 g/day) for 3 months and the placebo maltodextrin group as reference/clinical trials (30 obese women, BMI > 30 kg/m^2^, age range of 18 to 65 years) [207]		↑															*Bifidobacterium longum,* *B. pseudocatenulatum;* *B. adolescentis*	
Dewulf et al. (2013)	ITF (Synergy 1, inulin/ oligofructose 50/50 mix, 16 g/day) for 3 months and placebo (maltodextrin, 16 g/d) as reference/clinical trials (30 obese women, BMI > 30 kg/m^2^, age range of 18 to 65 years) [204]		↑			↑										↓		*Fecalibacterium prausnitzii;* *Bacteroides intestinalis;* *B. vulgatus*	
Ramirez-Farias et al. (2008)	Inulin (10 g/day) for 16 days and a control period without any supplement intake as reference/clinical trials (12 human volunteers) [203]		↑			↑													
Catry et al. (2017)	*n*-3 polyunsaturated fatty acid-depleted diet for 12 weeks with inulin-type fructans (ITF) (250 mg/mouse/d) for the last 15 days and group without ITF as reference/mice model (*n*-3 PUFA-depleted Apoe−/− mice) [202]				↑														No significant effect
Licht et al. (2006)	Inulin (150 g/kg) in diet for 5 weeks and baseline as reference/Rats model (8 rats with western type diet) [201]																↓		
High-fiber diet																			
Dao et al. (2016)	Calorie-restricted diet (enriched with fiber and protein) for 6 weeks and baseline as reference/clinical trials (49 overweight and obese adults) [167]				↑													*Akkermansia muciniphila*	
Candela et al. (2016)	Fiber-rich macrobiotic Ma-Pi 2 diet and a diet recommended by Italian professional societies for type 2 diabetes (T2 D) treatment, baseline as reference/clinical trials (40 overweight T2 D patients, aged 50–77 years) [168]				↑	↑	↑		↑										↑
Holscher et al. (2014)	Soluble corn fiber (21 g/day) for 21 days and no supplemental fiber placebo as reference/clinical trials (14 healthy adult men) [169]	↓							↑										
Tap et al. (2015)	A basal diet supplemented with dietary fiber (40 g/day) for 5 days and diet with dietary fiber (10 g/day) as reference/clinical trials (19 healthy adults: 9 males and 10 females, aged 19–25 years) [170]									↑									↑
Kovatcheva-Datchary et al. (2015)	Barley kernel-based bread consumption for 3 days and white wheat flour bread group as reference/clinical trials (39 healthy subjects: 6 men and 33 women, age 50–70 years, BMI 18–28 kg/m^2^) [171]									↑								*Prevotella copri*	
García-Peris et al. (2012)	Mixture of fiber (6 g twice daily, 50% inulin and 50% fructo-oligosaccharide) from one week before to three weeks after radiotherapy and the placebo (maltodextrin, 6 g twice daily) as reference/clinical trials (≥ 18-year-old female patients with gynecological cancer who received radiotherapy after surgery) [166]		↑	↑															
De Filippo et al. (2010)	High-fiber diet represented by the one of children in a rural African village of Burkina Faso (BF) and a diet represented by the one of European (EU) children as reference/clinical trials (15 healthy children in BF, additional 15 healthy children in EU, age range of 1–6 years) [172]	↓																	↑
Benus et al. (2010)	Dietary fiber formula (dietary fiber 19.6 and 18.0 g/day) for 14 days and the fiber-free diet as reference/clinical trials (10 healthy subjects: 6 women and 4 men, age range of 21–34 years) [164]		↑																
Carvalho-Wells et al. (2010)	Maize-derived whole grain cereal (48 g/day) for 21 days and the same dose of placebo cereal as reference/clinical trials (28 healthy volunteers: 7 males and 21 females, age range 20–51 years, BMI 20–30 kg/m^2^) [165]		↑																
Avila-Nava et al. (2017)	Pre-hispanic Mexican diet (PMD: containing corn flour, black beans concentrate, nopal, chia and pumpkin seed, with 8 g fiber/100 g diet) for 3 months and control diet (AIN-93) as reference/rats model (18 rats sucrose-enriched high-fat diet caused gut microbiota dysbiosis) [162]	↓		↑															
Resistant Starch																			
Martínez et al. (2010)	Crackers (100 g/day) containing native starch for 3 weeks and the baseline as references/clinical trials (10 subjects: age range of 23–38 years) [210]					↑												*Fecalibacterium prausnitzii*	
	Crackers (100 g/day) containing 33 g type 2 resistant starch (RS) for 3 wks and RS type 4 as references /Clinical trials (10 subjects: age range of 23–38 years) [210]							↑				↑						*Ruminococcus bromii Eubacterium rectale*	
	Crackers (100 g/d) containing 33 g type 4 RS for 3 weeks and the baseline as references/clinical trials (10 subjects: age range of 23–38 years) [210]	↓	↑															*Bifidobacterium adolescentis*	
Butyrylated Starch																			
Hald et al. (2016)	Resistant starch (type 2) and arabinoxylan enriched diet for 4 weeks and the baseline as reference/clinical trials (19 subjects: 39–75 years, with metabolic syndrome) [174]		↑																↓
	Resistant starch (type 2) and arabinoxylan enriched diet for 4 weeks and a low-fiber western-style diet group as reference/clinical trials (19 subjects: 39–75 years, with metabolic syndrome) [174]		↑																↓
Le Leu et al. (2015)	Butyrylated high-amylose maize starch (HAMSB, 40 g/d) with a high red meat diet (HRM, cooked red meat 300 g/d) for 4 weeks and baseline as reference/clinical trials (23 individuals with red meat-increased O6-methyl-2-deoxyguanosine adduct level) [212]			↑							↑	↑		↓				*Clostridium coccoides* *C. leptum* group *Ruminococcus bromii;* *R. torques; R. gnavus*	
Oligofructose																			
Wernimont et al. (2015)	Formula with oligofructose (OF, 3.0 g/L) for 8 weeks and identical formula without OF but enriched with α-lactalbumin (3.0 g/L) as reference/clinical trials (48 healthy infants) [214]		↑																
Cluny et al. (2015)	High-fat/high-sucrose (HFS) diet with 10% oligofructose for 6 weeks and HFS diet group as reference/rats model (46 rats with diet-induced obesity (23) and diet-resistant (23)) [213]		↑	↑			↑								↓			*Clostridium leptum*	
Polydextrose																			
Costabile et al. (2012)	Polydextrose (PDX; 8 g/d) for 3 weeks and the equivalent placebo (maltodextrin powder, 8 g/d) as reference/clinical trials (31 healthy subjects: age 18–50 years, BMI 19–25 kg/m^2^) [216]										↑	↑						*Ruminococcus intestinalis* *Clostridium* clusters I, II and IV	
High/low carbohydrate diet																			
Haro et al. (2016)	High-complex carbohydrate diet with low fat (28% fat, 12% monounsaturated) for 1 year and the baseline group as reference/clinical trials (20 obese patients (men) with coronary heart disease) [173]					↑	↓			↑								*Fecalibacterium prausnitzii*	
Ley et al. (2006)	Carbohydrate-restricted low-calorie diet for 1 year with obese people and a lean people group as reference/clinical trials (12 obese people) [35]	↓							↑										

Note: (↑): increased F/B or microbiota population; (↓): reduced F/B or microbiota population; Blank: not reported. The potentially beneficial microbiota and potentially detrimental microbiota listed in the form were based on the information from the literature of the reviewed studies, excluding the taxa without clear description of their function.

**Table 4:** Modulations of dietary fat on potentially beneficial and detrimental gut microbiota.

	Dose and Treatment Duration/Test Model	F/B	Potentially Beneficial Microbiota													Potentially Detrimental Microbiota										Species	Diversity
			*Bifidobacterium* sp.	*Lactobacillus* sp.	*Akkermansia* sp.	*Fecalibacterium* sp.	*Roseburia* sp.	*Eubacterium* sp.	*Bacteroides* sp.	*Prevotella* sp.	*Blautia* sp.	*Parabacteroides* sp.	*Ruminococcus* sp.	*Oscillospira* sp.	*Allobaculum* sp.	*Clostridium* sp.	*Enterococcus* sp.	*Escherichia* sp.	*Bilophila* sp.	*Streptococcus* sp.	*Alistipes* sp.	*Bacteroides* sp.	*Serratia* sp.	*Pantoea* sp.	*Citrobacter* sp.		
Saturated fat																											
Patterson et al. (2014)	High-palm oil (45% energy from fat) for 16 weeks and compared with low-fat diet (12% energy from fat)/mice model [235]	↑																									
De Wit et al. (2012)	High-palm oil (45% energy from fat) for 8 weeks and low-palm oil diet (10% energy from fat) as reference/mice model [236]	↑														↑										*Clostridium XI*	↓
Devkota et al. (2012)	Lard-based fat (37% energy from fat) for 24 weeks and low-fat diet (5% energy from fat) as reference/mice model [228]	↑																									
	Milk-derived fat (37% energy from fat) for 24 weeks and low-fat diet (5% energy from fat) as reference/mice model [228]	↓																	↑							*B. wadsworthia*	↓
Zentek et al. (2012)	Diet with medium-chain fatty acids uncoated (MCFA) for 4 weeks and the diet without MCFA as reference/piglets model [237]			↑				↑																		*L. johnsonii;* *L. amylovorus*	
Polyunsaturated fatty acid (PUFA)																											
Younge et al. (2017)	Early enteral supplementation with a high fat-PUFA blend of fish oil and safflower oil up to 10 weeks and the standard nutritional therapy as reference/clinical trial (premature infants with an enterostomy) [239]															↓		↓		↓			↓	↓	↓		↑
Patterson et al. (2014)	High flaxseed/fish oil (45% energy from fat) for 16 weeks and compared with a low-fat diet (12% energy from fat)/mice model [235]		↑												↑												↑
Devkota et al. (2012)	High safflower oil (37% energy from fat) for 24 weeks and low-fat diet (5% energy from fat) as reference/mice model [228]	↓																									↓
Fat diet (17% lard)																											
Avila-Nava et al. (2017)	HFD (17% lard fat and 7% soy oil) for 6 months and control diet (7% soy oil) as reference/rat model [162]	↑	↓	↓	↓																						
Fat diet (20–21.45% fat)																											
Wan et al. (2019)	Fat diet (20%) for 6 months and the baseline as reference/clinical trial (healthy young adults) [230]					↑					↑																
Lecomte et al. (2015)	HFD (21.45% fat) for 16 weeks and normal diet (12% fat) as reference/rat model [229]	↓		↓																							↑
Qiao et al. (2013)	HFD (21.45% mixed fat) for 8 weeks and normal diet (4.89% fat) as reference/mice model [233]																↑	↑								*Escherichia coli*	
Fat diet (28–35%)																											
Wan et al. (2019)	Moderate fat diet (30%) for 6 months and the baseline as reference/clinical trial (healthy young adults) [230]	↓																									
Haro et al. (2017)	Dietary intervention of < 30% total fat for 2 years and the baseline as reference/clinical trial (male patients with coronary heart disease, who are obese and also with severe metabolic disease) [231]	↓				↑			↑	↑						↓				↓							
Haro et al. (2017)	Dietary intervention of minimum 35% fat for 2 years and the baseline as reference/clinical trial (male patients with coronary heart disease) [231]	↓				↑	↑		↑	↑		↑	↑													*Parabacteroides distasonis* *Fecalibacterium prausnitzii*	
Haro et al. (2016)	Dietary intervention of 28% fat for 1 years and the baseline as reference/clinical trial (male obese patients with coronary heart disease) [173]						↓			↑																*Fecalibacterium prausnitzii*	
	Dietary intervention of 35% fat for 1 years and the baseline as reference/clinical trial (male obese patients with coronary heart disease) [173]						↑			↓		↑		↑												*Parabacteroides distasonis*	
Fat diet (40%)																											
Wan et al. (2019)	High-fat diet (40%) for 6 months and the baseline as reference/clinical trial (healthy young adults) [230]	↓				↓															↑	↑					
	High-fat diet (40%) for 6 months and the lower-fat diet (20%) as reference/clinical trial (healthy young adults) [230]	↓				↓					↓										↑	↑					↓
HFD (44–45% fat)																											
Chen et al. (2018)	HFD (45% fat) for 8 weeks and control diet (10% fat) as reference/mice model [220]	↑																									
Collins et al. (2016)	HFD (44% mixed fat) for 16 weeks and low-fat diet (10% fat) as reference/mice model [53]																										↓
Hamilton et al. (2015)	HFD (45% mixed fat) for 1, 3, 6 weeks and normal diet (13% fat) as reference/rat model [218]	↑																									↓
Murphy et al. (2010)	HFD (45% fat) for 8 weeks and low-fat diet (10% fat) as reference/mice model [221]	↑																									
Hildebrandt et al. (2009)	HFD (45% fat) for 1 month and standard diet (12% fat) as reference/mice model [219]	↑																									
HFD (60% fat)																											
Ojo et al. (2016)	HFD (60% fat) for 12 weeks and control diet (10% fat) as reference/mice model [238]		↓		↓																						
Cowan et al. (2014)	HFD (60% fat) for 10 weeks and control diet (12% fat) as reference/rat model [224]	↑	↑													↑										*Clostridium leptum*	
Mujico et al. (2013)	HFD (60% fat) for 19 weeks and maintenance diet (12% fat) as reference/mice model [225]	↑	↓																								
Lam et al. (2012)	HFD (60% mixed fat) for 8, 12 weeks and control diet (10% fat) as reference/mice model [222]	↑		↓																							↓
Kim et al. (2012)	HFD (60% fat) for 8 weeks and low-fat diet (10% fat) as reference/mice model [223]	↑																									
HFD (72% fat)																											
Cani et al. (2008)	HFD (72% fat) for 4 weeks and standard A04 diet as reference/mice model [226]	↑		↓																							
Ley et al. (2005)	HFD (72% fat) for 19 weeks and maintenance diet (12% fat) as reference/mice model [34]	↑																									

Note: (↑): increased F/B or microbiota population; (↓): reduced F/B or microbiota population; Blank: not reported. The potentially beneficial microbiota and potentially detrimental microbiota listed in the form were based on the information from the literature of the reviewed studies, excluding the taxa without clear description of their functions.

**Table 5:** Modulations of dietary protein on potentially beneficial and detrimental gut microbiota.

	Dose and Treatment Duration/Test Model	F/B	Potentially Beneficial Microbiota													Potentially Detrimental Microbiota										Species	Diversity
			*Bifidobacterium* sp.	*Lactobacillus* sp.	*Desulfovibrio* sp.	*Robinsoniella* sp.	*Barnesiella* sp.	*Allobaculum* sp.	*Akkermansia* sp.	*Roseburia* sp.	*Blautia* sp.	*Fecalibacterium* sp.	*Bacteroides* sp.	*Clostridium* sp.	*Enterorhabdus* sp.	*Clostridium* sp.	*Alloprevotella* sp.	*Haemophilus* sp.	*Ethanoligenens* sp.	*Klebsiella* sp.	*Porphyromonas* sp.	*Escherichia* sp.	*Turicibacter* sp.	*Staphylococcus* sp.	*Enterococcus* sp.		
Blend protein																											
Moreno-Pérez et al. (2018)	Protein group (20 g/day) for 10 weeks and control group (no protein) as reference/clinical trials [247]	↓								↓	↓																No significant effect
	Protein group (20 g//day) for 70 days and before protein treatment group as reference/clinical trials [247]	↓								↓	↓																No significant effect
Casein protein																											
Rist et al. (2014)	Casein-based group/CAS (85–335 g/kg) for 3 experimental periods (7 days/period) and Soybean meal-based group/SBM (85–335 g/kg) as reference/piglet model [243]		↓	↓																							↓
Soybean protein																											
Rist et al. (2014)	SBM group (85–335 g/kg) for 3 experimental periods (7 days/period) and the CAS (85–335 g/kg) as reference/piglet model [243]																										↑
Crude protein																											
Kostovcikova et al. (2019)	High animal protein-based diet (514 g/kg) for 3 weeks and control group (176 g/kg) as reference/mice model [245]								↑													↑		↑		*A. muciniphila*	↓
	High animal protein-based diet (514 g/kg) for 3 weeks and baseline as reference/mice model [245]																					↑		↑	↑	*Escherichia coli*	
	Animal protein-based control diet (176 g/kg) for 3 weeks and baseline as reference/mice model [245]																					↑	↑	↑	↑		
	High plant protein-based diet (500 g/kg) for 3 weeks and control group (173 g/kg) as reference/mice model [245]																					↑		↑		*Escherichia coli*	
	High plant protein-based diet (514 g/kg) for 3 weeks and baseline as reference/mice model [245]																					↑			↑	*Escherichia coli*	
	Plant protein-based control diet (176 g/kg) for 3 weeks and baseline as reference/mice model [245]																								↑		
Hang et al. (2012)	High protein group (crude protein 609 g/kg) for 28 days and dry commercial group (264 g/kg) as reference/dog model [254]																										↓
Lubbs et al. (2009)	High protein group (crude protein 50%) for 8 weeks and moderate-protein group (crude protein 30%) as reference/cat model [255]		↓													↑										*C. perfringens*	↑
Milk protein																											
Vidal-Lletjós et al. (2019)	Isocaloric diets with 53% protein for 3 days and the diets with 30% protein as reference/DDS-treated mice model [256]				↓								↑				↑	↑		↑							↓
	Isocaloric diets with 53% protein for 3 days and the diets with 14% protein as reference/DDS-treated mice model [256]				↓												↑	↑		↑							
	Isocaloric diets with 30% protein for 3 days and the diets with 14% protein as reference/DDS-treated mice model [256]												↓														↑
	Isocaloric diets with 53% protein for 6 days and the diets with 30% protein as reference/DDS-treated mice model [256]				↓							↓		↓												*Clostridium* XI	↓
	Isocaloric diets with 53% protein for 6 days and the diets with 14% protein as reference/DDS-treated mice model [256]														↑			↑									
	Isocaloric diets with 30% protein for 6 days and the diets with 14% protein as reference/DDS-treated mice model [256]												↑														
	Isocaloric diets with 53% protein for 21 days and the diets with 30% protein as reference/DDS-treated mice model [256]									↓		↓	↓					↑	↑	↑	↑						
	Isocaloric diets with 53% protein for 21 days and the diets with 14% protein as reference/DDS-treated mice model [256]												↓					↑	↑		↑						
	Isocaloric diets with 30% protein for 21 days and the diets with 14% protein as reference/DDS-treated mice model [256]									↑																	
Mung bean protein																											
Nakatani et al. (2018)	HFD-mung bean protein isolate (MPI) group (205.3 g MPI) for 4 weeks and HFD group (casein 200 g rather than MPI) as reference/mice model [242]	↓		↓																							
Seafood protein																											
Holm et al. (2016)	Seafood western diet (exchange casein with seafood powder) for 12 weeks and meat western diet (exchange casein with lean meat powder) as reference/mice model [244]			↓		↑	↑	↑	↓																		
Whey Protein																											
McAllan et al. (2014)	HFD-whey protein isolate (WPI) group (HFD with 20% WPI) for 21 weeks and HFD group as reference/mice model [241]		↑	↑	↑											↓											
Fried meats																											
Shen et al. (2010)	Fried meats (fry without oil at 300℃ for 15 min, then ground, frozen, and freeze-dried) for fermentation over 48 h./in vitro, human fecal sample [248]															↑										*Clostridium perfringens*	

Note: (↑): increased F/B or microbiota population; (↓): reduced F/B or microbiota population; Blank: not reported. The potentially beneficial microbiota and potentially detrimental microbiota listed in the form were based on the information from the literature of the reviewed studies, excluding the taxa without clear description of their functions.

## Supplementary Materials

The following are available online at https://www.mdpi.com/2072-6643/12/2/381/s1, Table S1: Specific search terms (MeSH terms) for collecting research articles from 2005 to 2019.
