# Supplementary material for: Role of Dietary Nutrients in the Modulation of Gut Microbiota: A Narrative Review
Source: Nutrients. 2020 Jan 31;12(2):381. doi: 10.3390/nu12020381 (PMC7071260; doi:10.3390/nu12020381)
Supplement: Supplementary file 1 [file nutrients-12-00381-s001.pdf]

Supplementary Table S1. Specific search terms (MeSH terms) for collecting research articles from 2005 to 2019.

**Micronutrients**		**Macronutrients**		
Polyphenols	Vitamins, Minerals, and Trace elements	Carbohydrates	Fats	Proteins
Micronutritents,<br>Polyphenols,<br>Anthocyanine,<br>Catechin,<br>Caffeic acid,<br>Chlorogenic Acid,<br>Daidzein,<br>Epicatechin,<br>Genistein,<br>Phloridzin,<br>Quercetin,<br>Epigallocatechin-3-gallate,<br>Resveratrol,<br>Fruit polyphenol,<br>Tea polyphenol,<br>Wine polyphenol	Micronutrients,<br>Vitamin,<br>Manganese,<br>Cyanocobalamine,<br>Tocopherol,<br>Retinoic acid<br>Calcium,<br>Magnesium,<br>Iron,<br>Phosphorus,<br>Zinc,<br>Selinium,<br>Iodine	Macronutrients,<br>Arabinoxylan,<br>Chitosan,<br>Galacto-Oligosaccharides,<br>Inulin,<br>Fructan,<br>Lactose,<br>Oligofructose,<br>Resistant starch,<br>High carbohydrate diet,<br>Low carbohydrate diet,<br>High fibre diet	Macronutrients,<br>High fat diet,<br>HFD,<br>Flaxseed,<br>Fish oil,<br>Palm oil,<br>Lard,<br>Safflower oil,<br>PUFA,<br>Low fat diet	Macronutrient,<br>High protein,<br>Protein,<br>Casein,<br>Soybean,<br>Crude protein,<br>Mung bean

The above MeSH terms were combined with the words below to narrow down the search:

AND Gut Microbiota AND/OR Human, Rat, in-vitro, NOT review.
